# Omega-3 polyunsaturated fatty acids favourably modulate cardiometabolic biomarkers in type 2 diabetes: a meta-analysis and meta-regression of randomized controlled trials

**DOI:** 10.1186/s12933-018-0740-x

**Published:** 2018-07-07

**Authors:** Lauren L. O’Mahoney, Jamie Matu, Oliver J. Price, Karen M. Birch, Ramzi A. Ajjan, Diane Farrar, Robyn Tapp, Daniel J. West, Kevin Deighton, Matthew D. Campbell

**Affiliations:** 1 Institute for Sport, Physical Activity & Leisure, Leeds Beckett University, Leeds, LS6 3QS UK; 2 AGADA Diabetes Education and Research Institute, Ljubljana, Slovenia; 3 Multidisciplinary Cardiovascular Research Centre, University of Leeds, Leeds, UK; 4 Bradford Institute for Health Research, Bradford Royal Infirmary, Bradford, UK; 5 Department of Health Sciences, University of York, York, UK; 6 Population Health Research Institute, St George’s, University of London, London, UK; 7 The Melbourne School of Population and Global Health, The University of Melbourne, Melbourne, Australia; 8 Institute of Cellular Medicine, Newcastle University, Newcastle, UK; 9 Present Address: Leeds Institute of Rheumatic and Musculoskeletal Medicine, University of Leeds, Leeds, UK

**Keywords:** Type 2 diabetes, Omega-3 polyunsaturated fatty acids, Eicosapentaenoic acid, Docosahexaenoic acid, Cardiovascular disease, Meta-analysis

## Abstract

**Background:**

Randomized controlled trials (RCTs) suggest that supplementation with omega-3 polyunsaturated fatty acids (n-3PUFAs) may favourably modify cardiometabolic biomarkers in type 2 diabetes (T2DM). Previous meta-analyses are limited by insufficient sample sizes and omission of meta-regression techniques, and a large number of RCTs have subsequently been published since the last comprehensive meta-analysis. Updated information regarding the impact of dosage, duration or an interaction between these two factors is therefore warranted. The objective was to comprehensively assess the effect of n-3PUFAs supplementation on cardiometabolic biomarkers including lipid profiles, inflammatory parameters, blood pressure, and indices of glycaemic control, in people with T2DM, and identify whether treatment dosage, duration or an interaction thereof modify these effects.

**Methods:**

Databases including PubMed and MEDLINE were searched until 13th July 2017 for RCTs investigating the effect of n-3PUFAs supplementation on lipid profiles, inflammatory parameters, blood pressure, and indices of glycaemic control. Data were pooled using random-effects meta-analysis and presented as standardised mean difference (Hedges g) with 95% confidence intervals (95% CI). Meta-regression analysis was performed to investigate the effects of duration of supplementation and total dosage of n-3PUFAs as moderator variables where appropriate.

**Results:**

A total of 45 RCTs were identified, involving 2674 people with T2DM. n-3PUFAs supplementation was associated with significant reductions in LDL [ES: − 0.10, (95% CI − 0.17, − 0.03); p = 0.007], VLDL (ES: − 0.26 (− 0.51, − 0.01); p = 0.044], triglycerides (ES: − 0.39 (− 0.55, − 0.24; p ≤ 0.001] and HbA1c (ES: − 0.27 (− 0.48, − 0.06); p = 0.010]. Moreover, n-3PUFAs supplementation was associated with reduction in plasma levels of TNF-α [ES: − 0.59 (− 1.17, − 0.01); p = 0.045] and IL-6 (ES: − 1.67 (− 3.14, − 0.20); p = 0.026]. All other lipid markers, indices of glycaemic control, inflammatory parameters, and blood pressure remained unchanged (p > 0.05).

**Conclusions:**

n-3PUFAs supplementation produces favourable hypolipidemic effects, a reduction in pro-inflammatory cytokine levels and improvement in glycaemia. Neither duration nor dosage appear to explain the observed heterogeneity in response to n-3PUFAs.

*Trial registration* This trial was registered at http://www.crd.york.ac.uk as CRD42016050802

**Electronic supplementary material:**

The online version of this article (10.1186/s12933-018-0740-x) contains supplementary material, which is available to authorized users.

## Background

Cardiovascular disease (CVD) is the leading cause of morbidity and mortality in people with type 2 diabetes (T2DM) [1]. While treatment for T2DM predominantly focuses on improving glycaemic control, lowering glucose only marginally reduces cardiovascular risk [2–6]. Conversely, targeted correction of clustered cardiometabolic biomarkers (e.g. lipid parameters, inflammatory markers, and blood pressure) have been shown to markedly reduce CVD risk and mortality in T2DM [7].

Such risk factors are highly amenable to dietary modification [8], and dietary habits account for a substantial proportion of CVD-related deaths [9]. Recent studies have identified suboptimal intake of omega-3 polyunsaturated fatty acids (n-3PUFAs) to be a key individual dietary component associated with premature cardiometabolic mortality [9]. Observational studies consistently report independent associations between high n-3PUFA intake and low cardiometabolic risk [10] and the pleiotropic effects of n-3PUFAs on cell functioning that affect blood lipids, inflammation, and endothelial function are well established [11, 12]. These epidemiologic studies, as well as in vitro and in vivo data, have prompted randomised controlled trials (RCTs) to determine whether n-3PUFA supplementation can modify cardiometabolic biomarkers. For example, data suggest that n-3PUFA may improve postprandial hypertriglyceridemia, hyperglycaemia, insulin secretion ability and endothelial function in patients with impaired glucose metabolism and coronary heart disease [13]. In T2DM supplementation with n-PUFA has been shown to improve glucose, waist circumference, and insulin and homeostatic model of insulin resistance (HOMA-IR) [14]. However, it has also recently been reported that n-3PUFA fail to exert beneficial effects on oxidative and inflammatory parameters [15], despite improvements in triglycerides [15]. Nor were improvements observed in coagulation, metabolic, and inflammatory status in well-controlled patients with atherosclerotic vascular disease and T2DM [16].

Whilst several meta-analyses have been performed in T2DM, variable degrees of benefit on these biomarkers have been reported [17–21]. Significant heterogeneity of effect between primary trials has been observed [19–21], which is likely to have arisen, at least in part, as a result of variable supplementation dosage and duration, either of which may modify the effects of n-3PUFAs on cardiometabolic biomarkers [22]. Moreover, previous meta-analyses are limited by statistical power and omission of meta-regression techniques which limits the ability to draw inferences regarding dosage, duration and the interaction of dosage and duration of n-3PUFA intake [17–20]. Furthermore, since publication of the most-comprehensive meta-analysis almost a decade ago [20], a considerable number of well-controlled RCTs have been published.

A primary goal of diabetes management is to establish effective adjunct treatments which act to reduce CVD risk [23]. Information which helps to comprehensively characterise the impact of n-3PUFAs on cardiometabolic biomarkers in people with T2DM is much needed, and could offer valuable insight into the therapeutic use of n-3PUFA supplementation. Therefore, the purpose of this review was to perform a meta-analysis and meta-regression of RCTs to provide the most contemporary and comprehensive assessment to date concerning the effects of n-3PUFAs on cardiometabolic biomarkers including lipid profiles, inflammatory parameters, blood pressure, and indices of glycaemic control in T2DM. We aimed to model whether duration, dosage, or an interaction of duration and dosage, influences biomarkers of interest, to identify treatments patterns that may yield the greatest therapeutic benefit.

## Methods

### Data sources and searches

This meta-analysis was conducted in accordance with PRISMA (Preferred Reporting Items for Systematic Review and Meta-analyses) guidelines [24] and prospectively registered. Databases were searched including PubMed and The Cochrane Library as well as MEDLINE, SPORTDiscus, PsycINFO and CINAHL, via EBSCOhost, by LLO, JM and KD up to the 13th July 2017. For search terms see Additional file 1: Table S1. No language or date of publication restrictions were applied during the literature search. Reference lists of eligible RCTs and review articles were also searched to identify additional relevant trials. Corresponding authors were contacted by e-mail and asked to provide data on two occasions where; (i) only the abstract or partial data was available; (ii) combined results had been reported for those with and without diabetes or those with and without other significant medical conditions.

### Eligibility criteria

**Fig. 1:**
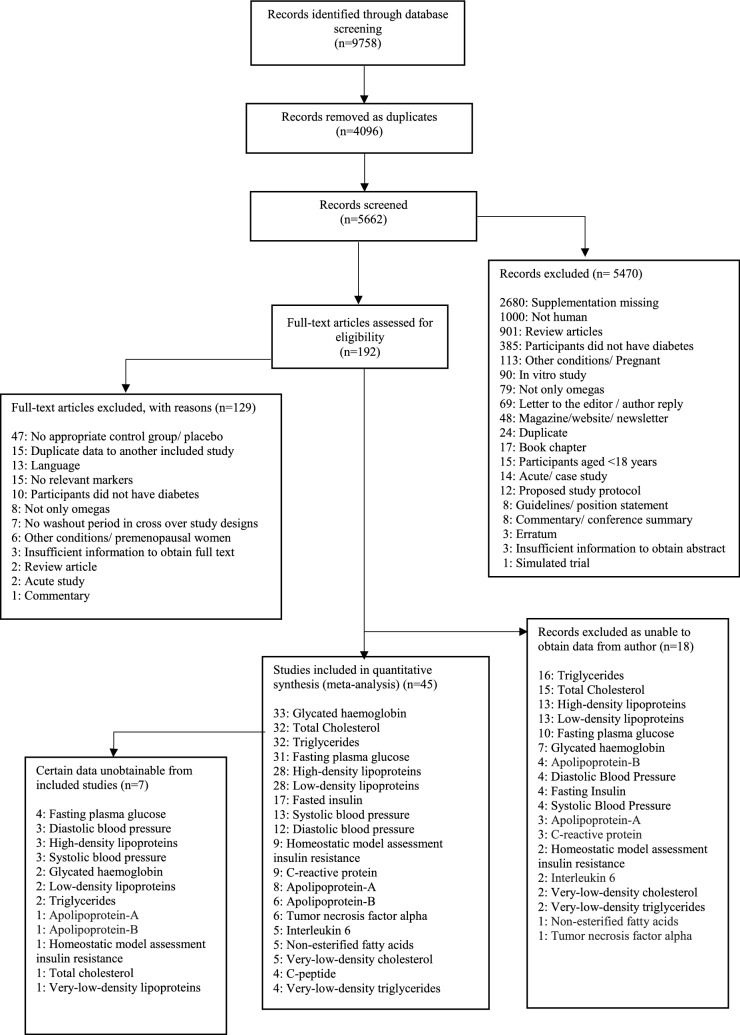
Flowchart of trial selection

Two reviewers (LLO and JM) independently reviewed all RCTs by title and abstract and subsequently by full text evaluation. Discrepancies which arose during this process were resolved by a third reviewer (KD). RCTs (either parallel or crossover designs) comparing the effects of n-3PUFAs with placebo control on outcomes of interest amongst adults with T2DM were included in the meta-analysis. All n-3PUFA interventions were in diet or capsule form and included if the dosage and duration could be determined. When RCTs assessed the effects of n-3PUFAs in conjunction with other nutrients or interventions, data were extracted from arms assessing n-3PUFA and placebo only. RCTs conducted in animal models, other forms of diabetes (e.g. type 1 diabetes, gestational diabetes), or in people under 18 years of age were excluded (Fig. 1 shows trial selection).

### Data extraction and quality assessment

Data extraction was conducted independently by two authors (LLO and MDC) with discrepancies adjudicated by a third author (KD). Data were extracted into a standardised spreadsheet, which included (i) trial information (first author, year of publication, corresponding author name and email); (ii) trial characteristics (design, number of trial arms, total duration, blinding); (iii) participant characteristics (gender, age, body mass, nationality, duration of diabetes and complications), and (iv) intervention specifics (type of n-3PUFA, placebo, duration, dosage, and number of participants per trial arm). Additionally, pre- and post-intervention mean and SD values were extracted, for: HbA1c (%, mmol/mol), fasting plasma glucose (FPG) (mmol L^−1^), fasting insulin (pmol L^−1^), HOMA-IR, C-peptide (nmol L^−1^), triglycerides (mmol L^−1^), total cholesterol (mmol L^−1^), high density lipoprotein (HDL) (mmol L^−1^), LDL (mmol L^−1^), very low density lipoprotein cholesterol (VLDL-C) (mmol L^−1^), very low density lipoprotein triglycerides (VLDL-TG) (mmol L^−1^), apolipoprotein-A1 (g L^−1^), apolipoprotein-B (g L^−1^), non-esterified fatty acids (NEFA) (mmol L^−1^/ng mL^−1^), C-reactive protein (CRP) (nmol L^−1^), tumour necrosis factor alpha (TNF-α) (pg mL^−1^), interleukin 6 (IL-6) (pg mL^−1^), systolic blood pressure (SBP) (mmHg), and diastolic blood pressure (DBP) (mmHg). When values were presented in figure form only, the figure was digitized using graph digitizer software (DigitizeIt, Germany) and the means and SD/SEM were measured manually at the pixel level to the scale provided.

Two independent reviewers (LLO and MDC) assessed the risk of bias in included trials using The Cochrane Collaboration’s tool for assessing risk of bias [25]. Each RCT was given one of three rankings, ‘high risk’, ‘low risk’, or ‘unclear risk’, in each of the following domains: sequence generation, allocation concealment, blinding of participants, personnel and outcome assessors, incomplete outcome data, selective outcome reporting and other sources of bias. Discrepancies which arose during this process were resolved firstly by discussion then by a third reviewer where necessary (KD). Risk of bias outcomes are presented within Additional file 1: Figures S1, S2.

### Data synthesis and analysis

If not reported, standard deviations were calculated from standard errors, confidence intervals (CI), or interquartile ranges [26]. Outcome measures were converted into the standardised mean difference (SMD) expressed as Hedges’ g with 95% CI. Correction using Hedges’ g is believed to yield an unbiased estimate of effect size [27]. A random-effects meta-analysis was performed [27] by LLO and KD using Comprehensive Meta-Analysis Software (Version 3, Biostat, Englewood, NJ, USA). The inputted data included sample sizes, outcome measures with their respective standard deviations, and a correlation coefficient for within-subject measurements for crossover designs. These correlation coefficients were estimated from prior trials in our laboratory and other published trials, and were as follows: HbA1c *r* = 0.90, fasted plasma glucose *r* = 0.62, fasted insulin *r* = 0.41, HOMA-IR *r* = 0.72, C-peptide *r* = 0.90, triglycerides *r* = 0.79, total cholesterol *r* = 0.90, HDL *r* = 0.60, LDL *r* = 0.89, VLDL-C *r* = 0.72, VLDL-TG *r* = 0.72, apolipoprotein-A1 *r* = 0.72, apolipoprotein-B *r* = 0.18, NEFA *r* = 0.30, CRP *r* = 0.81, TNF-α *r* = 0.94, IL-6 *r* = 0.90, SBP *r* = 0.80, DBP *r* = 0.80.

SMD values of < 0.20 were interpreted as trivial, 0.20–0.39 as small, 0.40–0.80 as moderate and > 0.80 as large [28]. A negative effect size (ES) favours n-3PUFA supplementation in the respective outcome variable while a positive ES favours the control. Heterogeneity between RCTs was assessed using the I^2^ statistic, where 0–20% suggests heterogeneity may be trivial, 20–50% represents low heterogeneity, 50–75% represent moderate heterogeneity, and 75% and above represents high heterogeneity [29]. This measure of heterogeneity was complimented by also reporting the Tau-squared statistic and the Chi squared statistic. To examine whether the results were affected markedly by a single trial, sensitivity analyses were performed on all outcome variables by iteratively omitting one trial at a time. Where significant effects of n-3PUFA on outcome measures were observed, post hoc meta-regression analysis (method-of-moments model) was performed where 10 or more trials were available to model the effect [27]. This analysis was used to determine whether duration, dosage, or both continuous moderator variables combined could explain the variation in ES between trials.

## Results

In total 5662 titles were found through database searches, of these 45 RCTs were eligible and included in the quantitative synthesis and meta-analysis (Fig. 1). A total of 2674 adults aged between 33 and 70 years with a T2DM diagnosis of between 1 and 19 years were included. The dose of total n-3PUFAs ranged from 0.40 to 18.00 g, with duration of supplementation lasting 2–104 weeks. n-3PUFAs were typically administered in capsule form, except on two occasions where a sardine-enriched diet or liquid form of n-3PUFA was administered. Of the 45 RCTs 69% (n = 31) investigated lipid and lipoprotein profiles, 42% (n = 19) inflammatory parameters and blood pressure, and 80% (n = 36) indices of glycaemic control. Primary outcomes of each study are provided (see Additional file 1: Table S7). Descriptive and raw data of included RCTs are provided: See Additional file 1: Tables S2–S5.

### Lipid and lipoprotein profiles

**Fig. 2:**
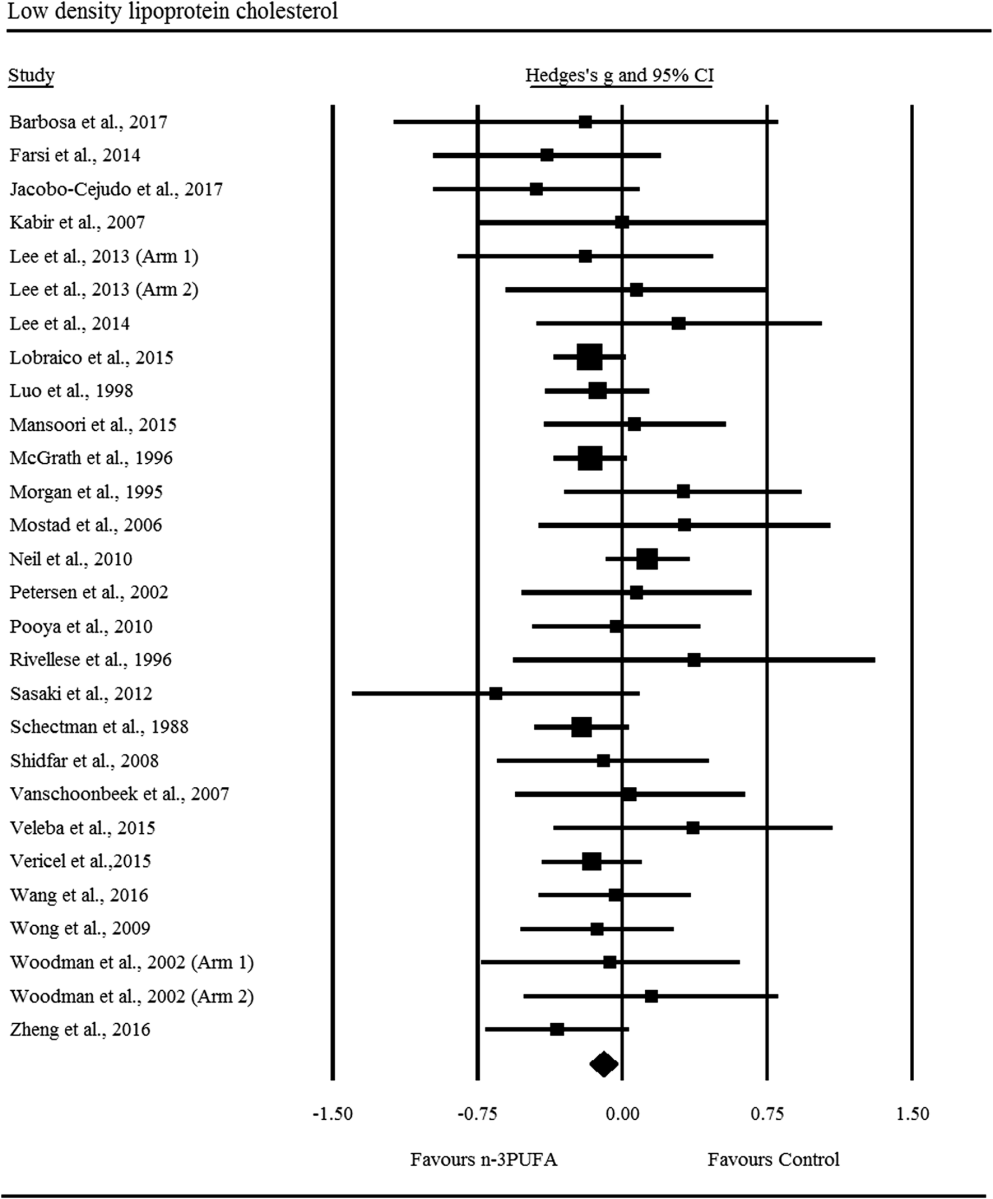
Forest plot of effect sizes (means ± 95% confidence intervals) for trials evaluating the effect of n-3PUFAs on low density lipoprotein cholesterol amongst adults with type 2 diabetes

**Fig. 3:**
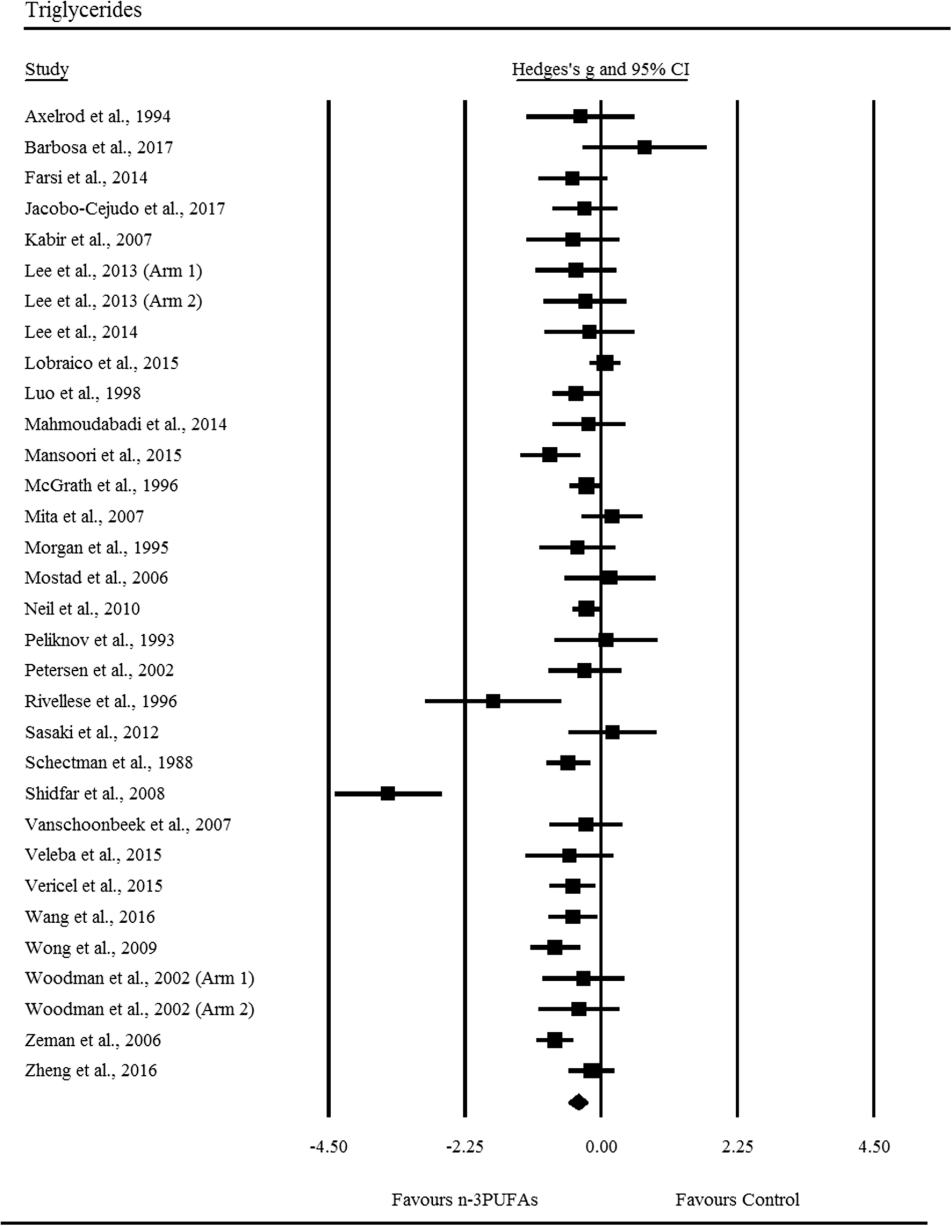
Forest plot of effect sizes (means ± 95% confidence intervals) for trials evaluating the effect of n-3PUFAs on triglycerides amongst adults with type 2 diabetes

**Fig. 4:**
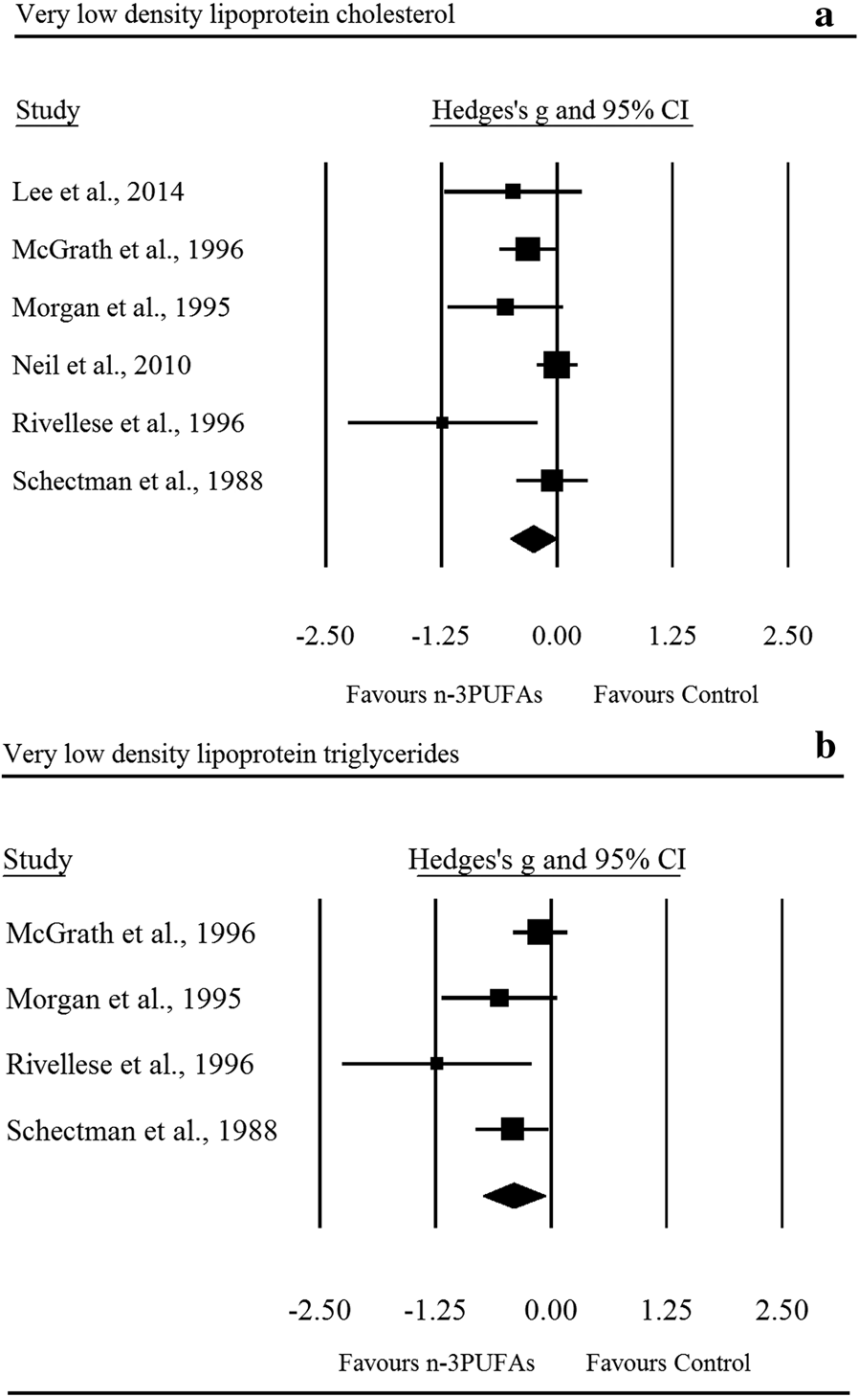
Forest plot of effect sizes (means ± 95% confidence intervals) for trials evaluating the effect of n-3PUFAs on very low density lipoprotein cholesterol (**a**) and very low density lipoprotein triglycerides (**b**) amongst adults with type 2 diabetes

Supplementation with n-3PUFAs resulted in a *trivial* decrease in LDL (ES: − 0.10, 95% CI − 0.17 to − 0.03; *p *= 0.007; Fig. 2), the degree of heterogeneity between these RCTs was also trivial (*I*^2^ = 0.00%; Q = 21.60, *τ*^2^ = 0.00, df = 27). There was a *small* significant decrease in triglycerides following n-3PUFA supplementation (ES: − 0.39, 95% CI − 0.55 to − 0.24; *p *≤ 0.001; Fig. 3), with moderate heterogeneity observed between RCTs (*I*^2^ = 69.40%, Q = 101.20, *τ*^2^ = 0.13, df = 31). n-3PUFAs were associated with a *small* significant decrease in VLDL-C (ES: − 0.26, 95% CI − 0.51 to − 0.01; *p *= 0.044; Fig. 4a), the degree of heterogeneity between these RCTs was low (*I*^2^ = 49.70%; Q = 9.90, *τ*^2^ = 0.04 and df = 5). Sensitivity analysis, performed to determine the independent effect of each trial on the overall effect size, revealed the independent removal of four single comparisons moderated the statistical interpretation of VLDL-C from significant to non-significant. There was a significant *moderate* reduction in VLDL-TG following n-3PUFA supplementation (ES: − 0.40, 95% CI − 0.74 to − 0.06; *p *= 0.021; Fig. 4b) the degree of heterogeneity between RCTs was low (*I*^2^ = 48.00%; Q = 5.80, *τ*^2^ = 0.06 and df = 3). The statistical interpretation of VLDL was changed from significant to non-significant by the independent removal of two RCTs. HDL (ES: − 0.09, 95% CI − 0.18 to 0.01; *p *= 0.067), total cholesterol (ES: − 0.03, 95% CI − 0.16 to 0.22; *p *= 0.733), NEFA (ES: − 0.96, 95% CI − 2.20 to 0.28; *p *= 0.128), apolipoprotein-A1 (ES: 0.03, 95% CI − 0.12 to 0.19; *p *= 0.656), and apolipoprotein-B (ES: 0.03, 95% CI − 0.25 to 0.30; *p *= 0.859) did not change significantly following n-3PUFA supplementation. Sensitivity analysis revealed that for HDL, the exclusion of three single comparisons in turn moderated the statistical interpretation of the results from non-significant to significant, resulting in a favourable increase in HDL following n-3PUFA supplementation compared to placebo.

### Inflammatory parameters and blood pressure

**Fig. 5:**
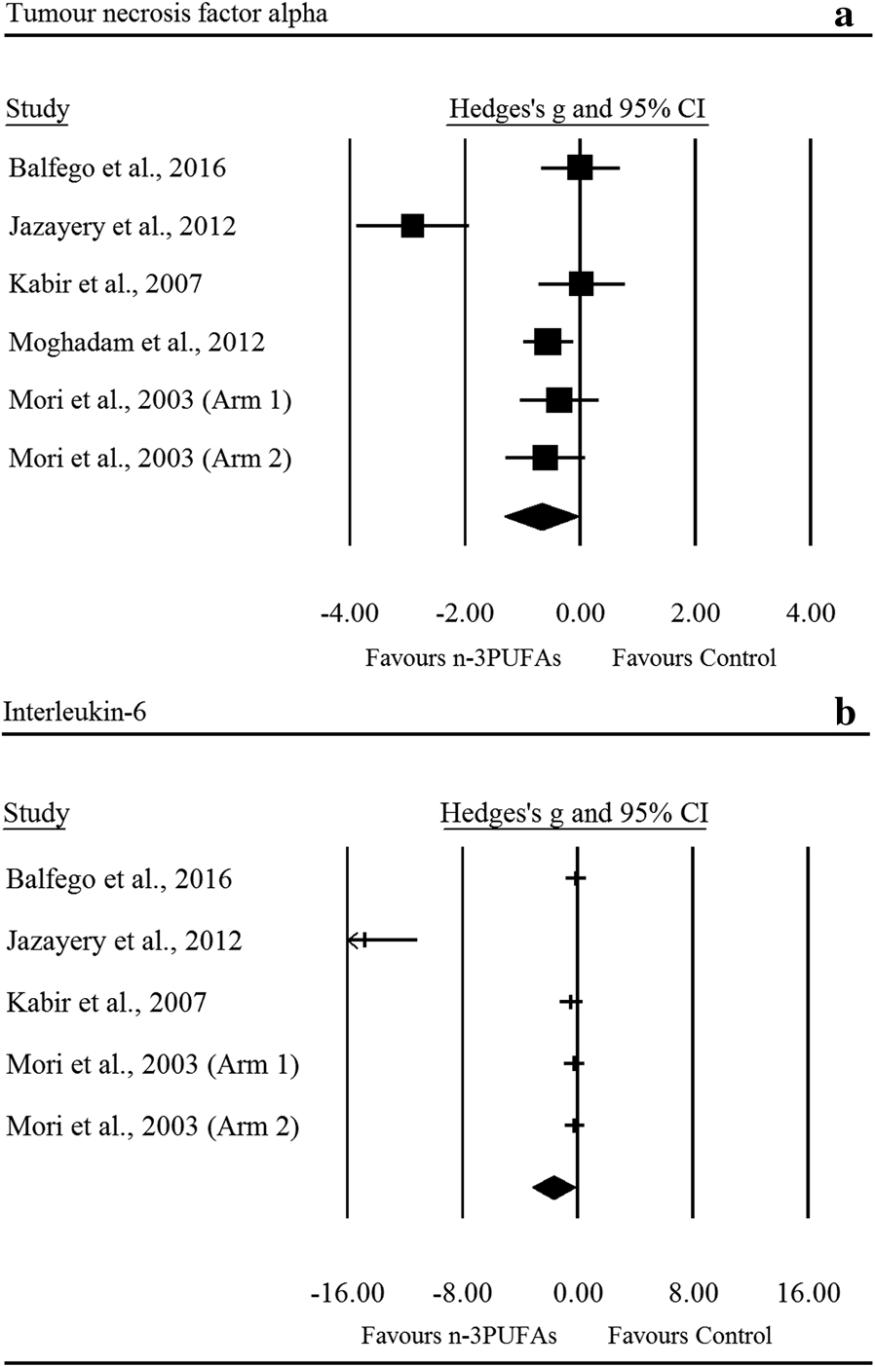
Forest plot of effect sizes (means ± 95% confidence intervals) for trials evaluating the effect of n-3PUFAs on TNF-α (**a**) and IL-6 (**b**) amongst adults with type 2 diabetes

There was a significant *moderate* reduction in TNF-α following n-3PUFA supplementation (ES: − 0.68, 95% CI − 1.32 to − 0.03; *p *= 0.039; Fig. 5a); the degree of heterogeneity was high between RCTs (I^2^ = 82.10%, Q = 27.90, τ^2^ = 0.52, df = 5). Sensitivity analysis for TNF-α revealed the removal of three single comparisons in turn moderated the statistical interpretation of the results from significant to non-significant. IL-6 was seen to decrease with a *large* ES (ES: − 1.67, 95% CI − 3.14 to − 0.20; *p *= 0.026; Fig. 5b); the degree of heterogeneity was high between RCTs (IL-6: I^2^ = 93.50%, Q = 61.60, τ^2^ = 2.35, df = 4). Sensitivity analysis for IL-6 revealed that the independent removal of one RCT moderated the statistical interpretation of the results from significant to non-significant. CRP did not change significantly with n-3PUFAs (ES: − 0.53, 95% CI − 1.28 to 0.21; *p *= 0.159), sensitivity analysis revealed that the removal of one comparison changed the results from non-significant to significant. n-3PUFAs had no significant effect on SBP (ES: 0.00, 95% CI − 0.15 to 0.14; *p *= 0.957) or DBP (ES: 0.04, 95% CI − 0.08 to 0.17; *p *= 0.508).

### Indices of glycaemic control

**Fig. 6:**
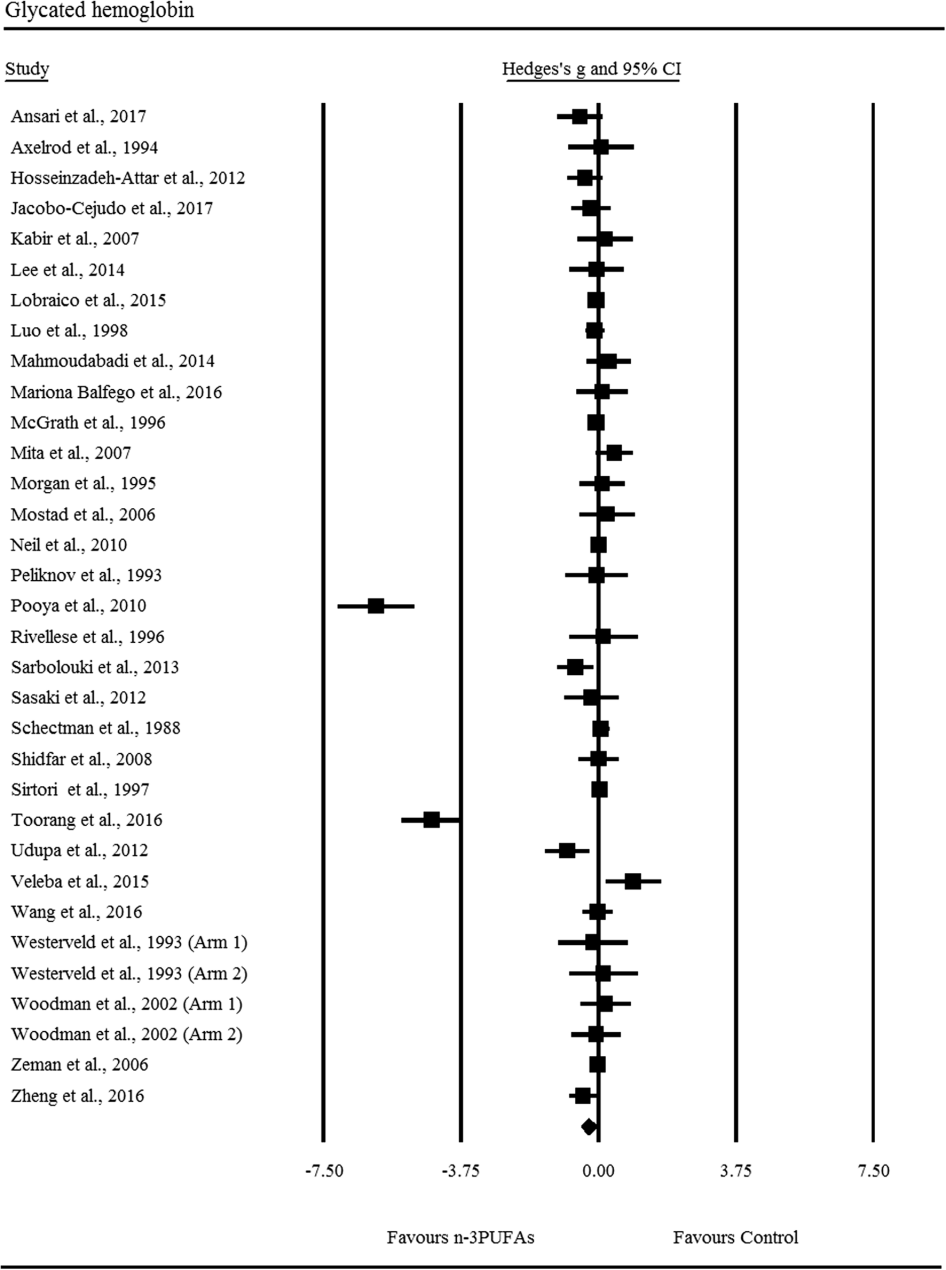
Forest plot of effect sizes (means ± 95% confidence intervals) for trials evaluating the effect of n-3PUFAs on HbA1c amongst adults with type 2 diabetes

There was a *small* yet significant reduction in HbA1c following n-3PUFA supplementation (ES: − 0.27, 95% CI − 0.48 to − 0.06; *p *= 0.010), the degree of heterogeneity was high between included RCTs (*I*^2^ = 88.60%, Q = 281.10, *τ*^2^ = 0.28, d_f_ = 32; Fig. 6). Sensitivity analysis for HbA1c revealed that the independent removal of two RCTs moderated the statistical interpretation of the results from significant to non-significant. All other indices of glycaemic control were not significantly different following n-3PUFA supplementation; FPG (ES: 0.07, 95% CI − 0.03 to 0.17; *p *= 0.177), fasting insulin (ES: − 0.12, 95% CI − 0.27 to 0.03; *p *= 0.105), HOMA-IR (ES: − 0.16, 95% CI − 0.37 to 0.05; *p *= 0.145), C-peptide (ES: 0.13, 95% CI − 0.14 to 0.41; *p *= 0.345).

### Meta regression

Meta-regressions were performed for significant outcomes with more than 10 RCTs; neither duration of supplementation nor dosage of n-3PUFAs statistically explained heterogeneity (i.e. variation in effect sizes) observed in these analyses (Table 1).

**Table 1:** Summary of meta-regression analysis using dosage and duration as covariates on appropriate cardiometabolic biomarkers (i.e. ≥ 10 RCTs)

Moderator variable	*p* value	Meta-regression of moderator variable vs. effect size
Low density lipoprotein		
Duration	0.85	Slope 0.00, 95% CI − 0.01 to 0.01, df = 27
Dosage	0.14	Slope 0.03, 95% CI − 0.01 to 0.07, df = 27
Triglycerides		
Duration	0.35	Slope 0.01, 95% CI − 0.01 to 0.01, df = 31
Dosage	0.87	Slope -0.00, 95% CI − 0.07 to 0.08, df = 31
Glycated haemoglobin		
Duration	0.11	Slope 0.01, 95% CI − 0.00 to 0.02, df = 32
Dosage	0.55	Slope 0.03, 95% CI − 0.07 to 0.13, df = 32

### Small study effects

Inspection of the funnel plots (Additional file 1: Figure S3) and Egger’s regression intercept revealed that there was little evidence of small study effects for triglycerides (intercept: − 0.78, 95% CI − 2.35 to 0.80; *p *= 0.32), LDL (intercept: − 0.49, 95% CI − 0.22 to 1.19; *p *= 0.17), TNF-α (intercept: − 3.50, 95% CI − 14.58 to 7.58; *p *= 0.43), and HbA1c (intercept: − 1.52, 95% CI − 3.40 to 0.36; *p *= 0.11). There was evidence of small study effects for VLDL-C (intercept: − 2.38, 95% CI − 4.39 to − 0.36; *p *= 0.03), VLDL-TG (intercept: − 2.89, 95% CI − 5.49 to − 0.30; *p *= 0.04), and IL-6 (intercept: − 9.63, 95% CI − 10.75 to − 8.50; *p *= <0.001).

## Discussion

Our meta-analysis and meta-regression provides the largest, most comprehensive, and contemporary review to date assessing the impact of n-3PUFAs on cardiometabolic biomarkers in T2DM. Considering the cumulative trial data from 45 pooled RCTs with a total of 2674 adults with T2DM, compared with placebo, n-3PUFA treatment was associated with significant hypolipidemic and anti-inflammatory effects, as well as a small but significant reduction in HbA1c. These improvements were not moderated by treatment duration, dosage, or an interaction between these two factors.

It is well established that T2DM is associated with dyslipidaemia (4). n-3PUFA intake has long been indicated in the treatment of hypertriglyceridemia [30, 31], promoting reductions in hepatic TG synthesis and accelerating triglyceride clearance [32–34]. We observed a small reduction in triglycerides in response to n-3PUFA intake accompanied by reductions in both VLDL-TG and VLDL-C which is largely consistent with previous findings [17–21]. However, contrary to previous meta-analyses [17–20], our analysis shows that n-3PUFA intake does not result in an unfavourable increase in LDL. This is an important observation, as LDL is an independent predictor of CVD risk, and treatment aimed at lowering LDL levels have shown CVD benefits and reduction in mortality [35, 36]; thus, LDL reduction is a principle target of primary prevention of CVD for the American College of Cardiology and the American Heart Association [37].

Previous research has highlighted, the magnitude of change in LDL is potentially dependent upon baseline triglyceride levels and may also differ between purified eicosapentaenoic acid (EPA)/docosahexaenoic acid (DHA) treatments and combined preparations [38]. We did not find improvements in total cholesterol, HDL, or apolipoproteins, which is consistent with previously published studies [18–20].

This is the first meta-analysis to show significant improvements in the inflammatory cytokines TNF-α and IL-6 in people with T2DM in response to n-3PUFA intake. Low grade inflammation is a pathologic mediator of vascular complications in T2DM [39], and the magnitude of cardiometabolic risk associated with plasma levels of acute-phase reactants [40, 41] is similar to that in isolated dyslipidaemia and hypertension [39, 42]. Epidemiological, cellular, and molecular data support T2DM as a state of amplified inflammation [43, 44]; which is associated with a heightened atherosclerotic and pro-thrombotic milieu [44], pathophysiologic insulin resistance [45, 46], and pancreatic cell apoptosis [47]. We observed reduction in plasma levels of the pro-inflammatory cytokine TNF-α as well as IL-6, in the absence of change to HOMA-IR and C-peptide. Thus, it is possible that n-3PUFAs exerts putative inflammatory-modifying effects which do not translate to improved insulin sensitivity or beta-cell function. Indeed, inflammatory signalling is complex and multifaceted and it is likely that distinct portions of the inflammatory signalling pathways may be affected differentially by n-3PUFAs. Our analysis included up to ~ threefold more studies than previous meta-analyses (IL-6: 2 vs. 5 RCTs; TNF-α: 2 vs. 6 RCTs) [20], and the results are consistent with the hypothesis that n-3PUFA exerts reductions in inflammatory markers.

Overall, we observed a small reduction in HbA1c following n-3PUFA supplementation. Earlier work has indicated that n-3PUFAs may result in adverse effects on HbA1c in patients with T2DM, from which increased basal hepatic glucose output and impaired insulin secretion are postulated to be responsible [48]. Our findings are contrary to previous meta-analyses assessing the use of n-3PUFAs on HbA1c in T2DM [18–21]. However, it is important to highlight that the overall ES for HbA1c is substantially increased by two RCTs and sensitivity analysis suggests the removal of either one of those trials changes the statistical interpretation of the test. Furthermore, we were unable to detect any effect of duration, dosage, or an interaction thereof, on HbA1c reduction. It is important to note however, that only 14 of the 33 RCTs included in this analysis were conducted ≥ 3 months, and that no effect was found on other indices of glycaemic control (FPG, fasting insulin, HOMA-IR, C-peptide). This highlights the requirement for longitudinal research to determine the effects of n-3PUFAs on glycaemic control in T2DM. In keeping with previous literature we found no significant changes in systolic or diastolic blood pressure following supplementation with n-3PUFAs [20], suggesting limited impact on vascular tone.

This meta-analysis and meta-regression provides the most comprehensive and contemporary review to date, assessing 19 cardiometabolic biomarkers, and including 45 RCTs—21 more than the largest aggregate data meta-analysis on this topic, and assessed whether treatment dosage, duration or an interaction thereof modify effects. By adopting a random-effects approach over fixed-effects to account for the true variation in effect size from trial to trial [27], and employing meta-regression techniques over subgroup analyses [49], our approach advances the findings from previous meta-analyses. Despite applying stringent inclusion criteria and rigorous methodology, some limitations must be acknowledged. Although no language restrictions were applied during the initial search, we were unable to translate 13 RCTs at full text stage which may have introduced language bias into the review. Sensitivity analyses for 5 outcomes of interest revealed that the removal of at least one trial moderated the statistical interpretation of the results from significant to non-significant. While the present meta-analysis had sufficient power to detect small effect sizes, smaller regression effects in some variables may have been lost as a result of the smaller number of trials due to the specific inclusion criteria. In addition, several concerns regarding the quality of the available evidence could be made, further high-quality evidence to support a beneficial effect of n-3PUFAs in T2DM patients could lead to more precise estimates of overall effect size. EPA and DHA may exert differential effects on cardiometabolic risk factors [50]. Due to the lack of qualifying studies investigating the independent effects of DHA (n = 4) and EPA (n = 9) on cardiometabolic risk factors, we were unable to differentiate between DHA, EPA, and concomitant administration. Quantifying the fatty acid composition of blood in n-3PUFA supplementation trials offers an objective measure of compliance and assessment of interindividual variability [51]. We encourage future research to include this, at least as a moderator variable, and to consider the influence of alternative assessment methods (i.e. plasma levels are indicative of acute intake whereas erythrocyte measurements reflect sustained intake) [52]. Although data relating to treatment adherence were not available for all studies, inclusion of non-adherent participants would bias results towards the null; thus, we can be confident that the effects of n-3PUFA in those who are fully adherent to supplementation will be no less than those reported for the study population overall. Although not possible in our meta-analysis, it would be of benefit to confirm findings on patient level data, which allow for predictors of supplementation outcome, and enable more precise studies in the future; other covariates such as duration of diabetes should be considered to see if they moderate the effects of n-3PUFAs. This meta-analysis intended to assess the effects of n-3PUFAs in both type 1 diabetes (T1DM) and T2DM, as originally outlined in the PROSPERO protocol. Unfortunately, only three RCTs investigating people with T1DM met the inclusion criteria (owing to inadequate experimental designs), highlighting the requirement for rigorously designed RCTs in this cohort. Considering T1DM presents with more severe permutations to the metabolic milieu compared to T2DM, it is not unreasonable to speculate that favourable findings from this analysis may translate and be more clinically relevant in the context of T1DM.

## Conclusions

Our study reports a major new indication for n-3PUFA intake: improvement in lipid profile and markers of inflammation without adverse effects on LDL or HbA1c. Neither duration of supplementation nor dosage of n-3PUFAs statistically explain the observed heterogeneity meaning that optimal treatment patterns for clinical practice are yet to be determined and further research is warranted. Future precision medicine trials should aim to establish whether interactions between n-3PUFAs and cardiometabolic biomarkers are modified by patient level characteristics to improve response to supplementation in T2DM and whether such improvements are observed in T1DM.

## Additional file

**Additional file 1: Table S1.** Predetermined search terms and strategy. **Table S2.** The effect of n-3 PUFAs on indices of glycaemic control. **Table S3.** The effect of n-3 PUFAs on lipid profiles. **Table S4.** The effect of n-3 PUFAs on Inflammatory parameters, and blood pressure. **Table S5.** The effect of n-3 PUFAs on apolipoproteins and non-esterified fatty acids. **Table S6.** Inclusion/exclusion criteria. **Table S7.** Primary outcome of included studies. **Figure S1.** Risk of bias across expressed as a percentage across all included studies. **Figure S2.** Risk of bias figure. **Figure S3 A-G.** Funnel plot of standard error by standard difference in means for LDL (A), TG (B), VLDL-C (C), VLDL-TG (D), TNF-α (E), IL-6 (F), and HbA1c (G). LDL, low density lipoprotein cholesterol; TG, triglycerides; VLDL-C, very low density lipoprotein cholesterol; VLDL-TG, very low density lipoprotein triglycerides; TNF-α, tumour necrosis factor alpha; IL-6, Interleukin 6; HbA1c, glycated haemoglobin.

## Abbreviations

CVD: cardiovascular disease
CI: confidence intervals
CRP: C-reactive protein
DBP: diastolic blood pressure
DHA: docosahexaenoic acid
EPA: eicosapentaenoic acid
ES: effect size
FPG: fasting plasma glucose
HbA1c: glycosylated haemoglobin
HDL: high density lipoprotein cholesterol
LDL: low-density lipoprotein cholesterol
n-3PUFAs: omega-3 polyunsaturated fatty acids
NEFA: non-esterified fatty acids
RCTs: randomised controlled trials
SBP: systolic blood pressure
SMD: standardised mean difference
T1DM: type 1 diabetes
T2DM: type 2 diabetes
VLDL-C: very low-density lipoprotein cholesterol
VLDL-TG: very low-density lipoprotein triglycerides
