# Supplementary material for: Omega-3 polyunsaturated fatty acids favourably modulate cardiometabolic biomarkers in type 2 diabetes: a meta-analysis and meta-regression of randomized controlled trials
Source: Cardiovasc Diabetol. 2018 Jul 7;17:98. doi: 10.1186/s12933-018-0740-x (PMC6035402; doi:10.1186/s12933-018-0740-x)
Supplement: Supplementary file 1 — Additional file 1: Table S1. Predetermined search terms and strategy. Table S2. The effect of n-3 PUFAs on indices of glycaemic control. Table S3. The effect of n-3 PUFAs on lipid profiles. Table S4. The effect of n-3 PUFAs on Inflammatory parameters, and blood pressure. Table S5. The effect of n-3 PUFAs on apolipoproteins and non-esterified fatty acids. Table S6. Inclusion/exclusion criteria. Table S7. Primary outcome of included studies. Figure S1. Risk of bias across expressed as a percentage across all included studies. Figure S2. Risk of bias figure. Figure S3 A-G. Funnel plot of standard error by standard difference in means for LDL (A), TG (B), VLDL-C (C), VLDL-TG (D), TNF-α (E), IL-6 (F), and HbA1c (G). LDL, low density lipoprotein cholesterol; TG, triglycerides; VLDL-C, very low density lipoprotein cholesterol; VLDL-TG, very low density lipoprotein triglycerides; TNF-α, tumour necrosis factor alpha; IL-6, Interleukin 6; HbA1c, glycated haemoglobin. [file 12933_2018_740_MOESM1_ESM.docx]

**Additional File**

| **Search string category** | **Search terms used** |
| --- | --- |
| (1) Diabetes | (diabetes) OR (diabetic) OR (NIDDM) OR (IDDM) OR (T1D) OR (T2D) OR (T1DM) OR (T2DM) |
| (2) EPA/DHA/ALA | (n-3 fatty acids) OR (n 3 fatty acids) OR (n-3 polyunsaturated fatty acid) OR (n 3 polyunsaturated fatty acid) OR (n-3 PUFA) OR (ω-3 fatty acids) OR (EPA) OR (eicosapentaenoic) OR (DHA) OR (alpha-linolenic acid) OR (ALA) OR (docosahexaenoic) OR (fish) OR (marine) |
| (3) Outcomes | ((HBA1c) OR (A1C) OR (A1c) OR (glycated haemoglobin) OR (glycosylated haemoglobin) OR (glucose) OR (glycerol) OR (insulin) OR (FPG) OR (PPG) OR (glycemic) OR (glycaemia) OR (glycemia) OR (fructosamine) OR (glycated albumin) OR (1,5,-anhydroglucitol) OR (1,5-AG) OR (glucose variability) OR (mean amplitude of glucose excursions) OR (MAGE) OR (mean daily differences) OR (MODD) OR (self-monitoring of blood glucose) OR (SMBG) OR (non-esterified fatty acids) OR (free fatty acids) OR (NEFA) OR (body mass index) OR (BMI) OR (body weight) OR (body mass) OR (cardiovascular) OR (cardioprotective) OR (cardioprotection) OR (cardiac) OR (lipaemia) OR (hyperlipemia) OR (lipemia) OR (lipidemia) OR (lipaemic) OR (dyslipidemia) OR (hyperlipidemia) OR (hypertriglyceridemia) OR (triglyceride) OR (triacylglycerol) OR (triacylglyceride) OR (TG) OR (TAG) OR (lipid) OR (lipoprotein) OR (cholesterol) OR (TC) OR (HDL) OR (LDL) OR (VDL) OR (chylomicrons) OR (apolipoprotein) OR (Inflammation) OR (inflammatory) OR (interleukin) or (IL-6) OR (IL-1) OR (tumor necrosis factor alpha) OR (TNF-alpha) OR (TNF-a) OR (cytokine) OR (c-reactive protein) OR (CRP) OR (blood pressure) OR (hypertension) OR (hypertensive) OR (antihypertensive) OR (endothelial) OR (endothelium) OR (coronary) OR (vessel) OR (vascular) OR (EPC) OR (EMP) OR (arteries) OR (arterial) OR (aortic) OR (antiatherogenic) OR (atherogenic) OR (arteriosclerosis) OR (atherosclerosis) OR (platelet) OR (flow-mediated dilation) OR (intima-media thickness)) |
| Combined search | (1) AND (2) AND (3) |

**Table S1.** Predetermined search terms and strategy.

**Table S2.** The effect of n-3 PUFAs on indices of glycaemic control.

| **Study** | **Intervention** | | | | **Outcomes** | | | | | | |
| --- | --- | --- | --- | --- | --- | --- | --- | --- | --- | --- | --- |
|  | **Design** | **Duration** | **Dosage** | **N** |  | **HbA1c** | **HbA1c** | **FPG** | **Insulin** | **HOMA-IR** | **C-peptide** |
|  |  | (Weeks) | (grams) | (exp vs con) |  | (%) | (mmol.mol^-1^) | (mmol.L^-1^) | (pmol.L^-1^) |  | (nmol.L^-1^) |
| Ansari et al., 2017 | Parallel | 10 | 1.80 EPA+0.90 DHA | 43 (22 vs. 21) | Exp Pre | 7.68±1.17 | 60.00±12.80 | - | 72.30±25.42 | 4.57±2.25 | - |
|  |  |  |  |  | Exp Post | 7.10±1.03 | 54.00±11.30 |  | 70.98±28.68 | 4.09±2.35 |  |
|  |  |  |  |  | Con Pre | 7.72±1.00 | 61.00±10.90 |  | 85.08±28.96 | 5.30±1.79 |  |
|  |  |  |  |  | Con Post | 7.64±0.90 | 60.00±9.80 |  | 86.95±28.61 | 5.62±2.52 |  |
| Axelrod et al., 1994 | Parallel | 6 | 1.55 EPA + 1.05 DHA | 18 (9 vs. 9) | Exp Pre | 7.60±0.90 | 60.00±9.80 | - | - | - | - |
|  |  |  |  |  | Exp Post | 7.64±0.70 | 60.00±7.77 |  |  |  |  |
|  |  |  |  |  | Con Pre | 7.40±1.20 | 57.00±13.10 |  |  |  |  |
|  |  |  |  |  | Con Post | 7.37±1.06 | 57.00±11.06 |  |  |  |  |
| Azizi-Soleiman et al., 2002 (Arm 1) | Parallel | 12 | 0.98 EPA | 31 (14 vs. 17) | Exp Pre | - | - | 5.10±0.80 | - | - | - |
|  |  |  |  |  | Exp Post |  |  | 5.60±0.80 |  |  |  |
| (Arm 2) | Parallel | 12 | 0.98 DHA | 31 (14 vs. 17) | Exp Pre | - | - | 5.10±0.90 |  |  |  |
|  |  |  |  |  | Exp Post |  |  | 5.70±0.80 |  |  |  |
|  |  |  |  |  | Con Pre |  |  | 5.60±0.90 |  |  |  |
|  |  |  |  |  | Con Post |  |  | 5.40±0.90 |  |  |  |
| Balfegó et al., 2016 | Parallel | 26 | 3.00 ± 0.20 (NS) | 32  (17 vs. 15) | Exp Pre | 6.86±0.44 | 51.00±4.80 | 7.67±1.57 | 119.45±51.46 | - | - |
|  |  |  |  |  | Exp Post | 6.60±0.41 | 49.00±4.50 | 7.14±1.01 | 77.09±51.53 |  |  |
|  |  |  |  |  | Con Pre | 6.70±0.40 | 50.00±4.40 | 7.45±1.13 | 104.87±50.00 |  |  |
|  |  |  |  |  | Con Post | 6.40±0.39 | 46.00±4.30 | 7.17±1.16 | 81.26±64.52 |  |  |
| Barbosa et al., 2017* | Parallel | 8 | 1.11 EPA+ 0.69 EPA | 14 (6 vs. 8) | Exp Pre | - | - | 5.45±0.94 | 85.28±16.39 | 3.02±0.90 | - |
|  |  |  |  |  | Exp Post |  |  | 6.24±2.44 | 94.24±19.10 | 3.85±1.84 |  |
|  |  |  |  |  | Con Pre |  |  | 8.53±3.26 | 139.87±104.80 | 7.36±4.76 |  |
|  |  |  |  |  | Con Post |  |  | 7.82±2.61 | 162.10±79.87 | 8.10±4.29 |  |
| Farsi et al., 2014 | Parallel | 10 | 1.40 EPA + 0.90 DHA | 44  (22 vs. 22) | Exp Pre | - | - | - | 74.17±27.78 | 4.15±2.20 | - |
|  |  |  |  |  | Exp Post |  |  |  | 59.10±19.45 | 3.07±1.60 |  |
|  |  |  |  |  | Con Pre |  |  |  | 52.78±19.45 | 3.15±2.00 |  |
|  |  |  |  |  | Con Post |  |  |  | 52.16±18.06 | 3.00±1.50 |  |
| Hoseinzadeh-Attar et al., 2012 | Parallel | 8 | 0.72 EPA + 0.48 DHA | 71 (37 vs. 34) | Exp Pre | 8.40±1.50 | 68.00±16.40 | 8.10±2.86 | - | - | - |
|  |  |  |  |  | Exp Post | 7.80±1.50 | 62.00±16.40 | 8.30±2.91 |  |  |  |
|  |  |  |  |  | Con Pre | 8.50±1.70 | 69.00±18.60 | 7.93±3.20 |  |  |  |
|  |  |  |  |  | Con Post | 8.50±1.70 | 69.00±18.60 | 8.35±3.07 |  |  |  |
| Jacobo-Cejudo et al., 2017 | Parallel | 26 | 0.30 EPA + 0.20 DHA | 54 (29 vs. 25) | Exp Pre | 9.60±3.10 | 81.00±33.90 | 9.83±3.80 | 52.78±20.84 | 3.10±1.30 | - |
|  |  |  |  |  | Exp Post | 8.20±1.90 | 66.00±20.80 | 8.66±3.85 | 98.62±56.95 | 5.30±3.80 |  |
|  |  |  |  |  | Con Pre | 10.00±2.10 | 86.00±23.00 | 10.25±3.95 | 45.14±11.11 | 2.90±1.20 |  |
|  |  |  |  |  | Con Post | 9.00±1.80 | 75.00±19.70 | 10.17±2.96 | 70.84±22.92 | 4.40±1.60 |  |
| Kabir et al., 2007 | Parallel | 8 | 1.08 EPA + 0.72 DHA | 26 (12 vs. 14) | Exp Pre | 7.30±0.70 | 56.00±7.70 | 8.20±2.42 | - | - | - |
|  |  |  |  |  | Exp Post | 7.40±1.12 | 57.00±12.20 | 8.00±2.42 |  |  |  |
|  |  |  |  |  | Con Pre | 7.80±1.50 | 62.00±16.40 | 8.90±2.99 |  |  |  |
|  |  |  |  |  | Con Post | 7.70±1.12 | 61.00±12.20 | 9.20±2.99 |  |  |  |
| Lee et al., 2014* | Parallel | 8 | 3.58 EPA + 2.44 DHA | 27  (13 vs. 14) | Exp Pre | 7.75±1.22 | 61.00±13.30 | 7.84±3.91 | 144.53±135.01 | 9.33±11.66 | - |
|  |  |  |  |  | Exp Post | 7.54±1.18 | 59.00±12.90 | 7.18±2.58 | 190.64±421.35 | 8.97±9.86 |  |
|  |  |  |  |  | Con Pre | 7.80±1.52 | 62.00±16.60 | 8.02±2.66 | 129.32±96.74 | 6.14±4.00 |  |
|  |  |  |  |  | Con Post | 7.67±1.62 | 60.00±17.70 | 8.06±3.50 | 120.57±70.08 | 5.84±3.84 |  |
| Lobraico et al., 2015 | Cross Over | 4 (2 WO) | 1.00 (NS) | 47 | Exp | 7.60±1.50 | 60.00±16.40 | 7.96±2.72 | - | - | 0.30±0.16 |
|  |  |  |  |  | Con | 7.70±1.60 | 61.00±18.60 | 8.10±3.18 |  |  | 0.31±0.14 |
| Luo et al., 1998 | Cross Over | 8 (8 WO) | 1.08 EPA + 0.72 DHA | 10 | Exp | 8.70±1.58 | 72.00±17.30 | 11.08±3.16 | 83.00±22.14 | - | - |
|  |  |  |  |  | Con | 8.90±1.90 | 74.00±20.80 | 11.23±3.79 | 76.00±31.62 |  |  |
| Mahmoudabadi et al., 2014 | Parallel | 8 | 0.50 EPA | 41 (21 vs. 20) | Exp Pre | 7.70±0.90 | 61.00±9.80 | 8.85±2.81 | - | - | - |
|  |  |  |  |  | Exp Post | 7.00±1.20 | 53.00±13.10 | 7.24±2.05 |  |  |  |
|  |  |  |  |  | Con Pre | 8.10±1.60 | 65.00±17.50 | 8.64±2.75 |  |  |  |
|  |  |  |  |  | Con Post | 7.10±0.90 | 54.00±9.80 | 7.50±2.75 |  |  |  |
| Mansoori et al., 2015 | Parallel | 8 | 0.40 EPA + 1.45 DHA | 68 (35 vs. 33) | Exp Pre | - | - | 10.09±3.66 | 56.95±38.20 | 3.50±2.60 | - |
|  |  |  |  |  | Exp Post |  |  | 10.06±3.52 | 50.00±29.17 | 3.10±2.00 |  |
|  |  |  |  |  | Con Pre |  |  | 9.27±2.79 | 58.34±35.42 | 3.50±2.50 |  |
|  |  |  |  |  | Con Post |  |  | 9.18±3.21 | 47.92±22.92 | 2.80±1.80 |  |
| McGrath et al., 1996 | Cross Over | 6 (6 WO) | 1.80 EPA + 1.60 DHA | 23 | Exp | 9.90±3.24 | 85.00±35.40 | 11.40±4.16 | - | - | - |
|  |  |  |  |  | Con | 9.70±2.77 | 83.00±30.30 | 11.00±3.93 |  |  |  |
| Mita et al., 2007 | Parallel | 104 | 1.80 EPA | 60 (30 vs. 30) | Exp Pre | 6.70±1.39 | 50.00±15.20 | - | - | - | - |
|  |  |  |  |  | Exp Post | 6.97±1.17 | 53.00±12.80 |  |  |  |  |
|  |  |  |  |  | Con Pre | 6.76±1.35 | 50.00±14.80 |  |  |  |  |
|  |  |  |  |  | Con Post | 6.56±0.98 | 48.00±10.70 |  |  |  |  |
| Morgan et al., 1995 | Parallel | 12 | 2.59-5.18 EPA + 2.46-4.91 DHA | 40 (20 vs. 20) | Exp Pre | 7.30±1.50 | 56.00±16.40 | 10.40±3.40 | - | - | - |
|  |  |  |  |  | Exp Post | 7.70±1.70 | 61.00±18.60 | 11.60±3.2 |  |  |  |
|  |  |  |  |  | Con Pre | 7.60±1.70 | 60.00±18.60 | 11.60±3.50 |  |  |  |
|  |  |  |  |  | Con Post | 7.80±2.00 | 62.00±21.90 | 12.40±3.50 |  |  |  |
| Mostad et al., 2006 | Parallel | 8 | 1.80 EPA + 3.00 DHA | 26 (12 vs. 14) | Exp Pre | 6.80±0.47 | 51.00±5.10 | 7.80±1.49 | 167.00±111.51 | - | 1.20±0.87 |
|  |  |  |  |  | Exp Post | 6.90±0.71 | 52.00±7.80 | 8.1±1.61 | 160.00±111.51 |  | 1.20±0.87 |
|  |  |  |  |  | Con Pre | 7.00±1.04 | 53.00±11.40 | 8.1±2.90 | 132.00±79.38 |  | 0.80±0.50 |
|  |  |  |  |  | Con Post | 6.90±0.87 | 52.00±9.50 | 8.1±3.02 | 132.00±70.56 |  | 0.80±0.50 |
| Neil et al., 2010 | Parallel | 17 | 1.68 EPA + 0.92 DHA | 326  (160 vs. 166) | Exp Pre | 7.00±1.10 | 53.00±12.00 | - | - | - | - |
|  |  |  |  |  | Exp Post | 7.10±1.10 | 54.00±12.00 |  |  |  |  |
|  |  |  |  |  | Con Pre | 6.90±1.10 | 52.00±12.00 |  |  |  |  |
|  |  |  |  |  | Con Post | 7.00±1.10 | 53.00±12.00 |  |  |  |  |
| Peliknov et al., 1993 | Parallel | 4 | 3.10 (NS) | 20 (10 vs. 10) | Exp Pre | 9.10±1.50 | 76.00±16.40 | 8.71±2.29 | - | - | - |
|  |  |  |  |  | Exp Post | 8.40±1.30 | 68.00±14.20 | 8.65±2.08 |  |  |  |
|  |  |  |  |  | Con Pre | 8.15±1.60 | 66.00±17.50 | 8.06±2.40 |  |  |  |
|  |  |  |  |  | Con Post | 7.50±0.59 | 58.00±6.40 | 7.45±2.00 |  |  |  |
| Pooya et al., 2010 | Parallel | 8 | 1.60 EPA + 0.80 DHA | 81 (40 vs. 41) | Exp Pre | 7.90±1.20 | 63.00±13.10 | 7.65±2.69 | - | - | - |
|  |  |  |  |  | Exp Post | 7.15±0.17 | 55.00±1.90 | 7.75±1.47 |  |  |  |
|  |  |  |  |  | Con Pre | 7.64±0.98 | 60.00±10.70 | 7.17±1.57 |  |  |  |
|  |  |  |  |  | Con Post | 7.90±0.16 | 63.00±1.70 | 7.00±1.90 |  |  |  |
| Rivellese et al., 1996 | Parallel | 26 | 1.00 EPA + 1.60 DHA | 16 (8 vs. 8) | Exp Pre | 7.30±1.13 | 56.00±12.40 | 10.20±1.20 | 75.00±9.00 | - | - |
|  |  |  |  |  | Exp Post | 8.30±1.41 | 67.00±15.40 | 10.90±0.50 | 105.60±15.60 |  |  |
|  |  |  |  |  | Con Pre | 6.90±1.41 | 52.00±15.40 | 9.20±0.60 | 121.00±18.40 |  |  |
|  |  |  |  |  | Con Post | 7.70±1.41 | 61.00±15.40 | 10.30±1.00 | 135.00±14.00 |  |  |
| Sarbolouki et al., 2013 | Parallel | 12 | 1.90 EPA | 67  (32 vs. 35) | Exp Pre | 8.92±1.39 | 74.00±15.20 | 8.13±2.15 | 50.98±21.25 | 2.73±1.31 | - |
|  |  |  |  |  | Exp Post | 8.14±1.22 | 65.00±13.30 | 7.05±1.77 | 46.95±16.04 | 2.10±1.73 |  |
|  |  |  |  |  | Con Pre | 8.91±1.81 | 74.00±19.80 | 10.38±3.12 | 37.23±15.14 | 2.43±1.76 |  |
|  |  |  |  |  | Con Post | 9.11±1.79 | 76.00±19.60 | 10.64±3.23 | 47.23±18.54 | 3.10±1.96 |  |
| Sasaki et al., 2012 | Parallel | 26 | 1.80 EPA | 28 (15 vs. 13) | Exp Pre | 6.70±0.90 | 50.00±9.80 | 7.33±2.22 | - | - | - |
|  |  |  |  |  | Exp Post | 6.80±1.00 | 51.00±10.90 | 7.88±1.89 |  |  |  |
|  |  |  |  |  | Con Pre | 6.70±0.60 | 50.00±6.60 | 7.22±1.50 |  |  |  |
|  |  |  |  |  | Con Post | 7.00±1.00 | 53.00±10.90 | 7.77±1.44 |  |  |  |
| Schectman et al., 1988 | Cross Over | 4 (4 WO) | 2.60 EPA + 1.40 DHA | 13 | Exp | 8.50±1.00 | 69.00±10.90 | 7.77±1.80 | - | - | 0.01±0.00 |
|  |  |  |  |  | Con | 8.40±1.33 | 68.00±14.50 | 7.83±2.00 |  |  | 0.01±0.00 |
| Shidfar et al., 2008 | Parallel | 10 | 0.50 EPA + 0.50 DHA | 50 (25 vs. 25) | Exp Pre | 7.40±0.20 | 57.00±2.20 | 8.23±1.68 | 115.90±64.00 | - | - |
|  |  |  |  |  | Exp Post | 7.30±0.30 | 56.00±3.30 | 8.42±1.71 | 119.70±84.50 |  |  |
|  |  |  |  |  | Con Pre | 7.10±0.10 | 54.00±1.10 | 8.11±1.54 | 111.60±57.20 |  |  |
|  |  |  |  |  | Con Post | 7.00±0.10 | 53.00±1.10 | 7.90±1.67 | 119.70±84.50 |  |  |
| Sirtori et al., 1997 | Parallel | 26 | 1.53 EPA + 1.05 DHA†/ 1.02 EPA + 0.70 DHA‡ | 414  (203 vs. 211) | Exp Pre | 7.25±1.56 | 56.00±17.10 | 8.26±2.17 | 115.90±64.00 | - | - |
|  |  |  |  |  | Exp Post | 7.05±1.64 | 54.00±17.90 | 8.16±2.05 | 112.00±50.70 |  |  |
|  |  |  |  |  | Con Pre | 7.14±1.63 | 55.00±17.80 | 8.14±2.06 | 111.60±57.20 |  |  |
|  |  |  |  |  | Con Post | 6.89±1.42 | 52.00±15.50 | 7.93±2.05 | 119.70±84.50 |  |  |
| Toorang et al., 2016 | Parallel | 8 | 1.55 EPA + 0.83 DHA | 81 (41 vs. 40) | Exp Pre | 7.90±0.20 | 63.00±2.20 | - | - | - | - |
|  |  |  |  |  | Exp Post | 7.25±0.17 | 56.00±1.90 |  |  |  |  |
|  |  |  |  |  | Con Pre | 7.64±0.20 | 60.00±2.20 |  |  |  |  |
|  |  |  |  |  | Con Post | 7.84±0.20 | 62.00±2.20 |  |  |  |  |
| Udupa et al., 2012 | Parallel | 13 | 1.08 EPA + 0.72 DHA | 43  (22 vs. 21) | Exp Pre | 11.36±1.61 | 100.00±17.60 | 7.89±1.20 | - | - | - |
|  |  |  |  |  | Exp Post | 9.53±1.30 | 81.00±14.20 | 7.73±1.16 |  |  |  |
|  |  |  |  |  | Con Pre | 11.18±1.45 | 98.00±15.80 | 7.88±1.23 |  |  |  |
|  |  |  |  |  | Con Post | 10.77±1.94 | 93.00±21.20 | 8.15±1.33 |  |  |  |
| Veleba et al., 2015 | Parallel | 24 | 0.75 EPA + 2.00 DHA | 29  (16 vs. 13) | Exp Pre | 8.20±1.00 | 66.00±10.90 | 7.69±1.89 | - | - | - |
|  |  |  |  |  | Exp Post | 9.70±2.60 | 83.00±28.40 | 8.98±2.55 |  |  |  |
|  |  |  |  |  | Con Pre | 8.40±1.00 | 68.00±10.90 | 7.33±1.55 |  |  |  |
|  |  |  |  |  | Con Post | 8.00±0.30 | 64.00±3.30 | 7.14±1.81 |  |  |  |
| Wang et al., 2016 | Parallel | 26 | 1.34 EPA + 1.07 DHA | 99  (49 vs. 50) | Exp Pre | 7.72±1.23 | 61.00±13.40 | 8.04±2.09 | 48.34±74.38 | 2.24±4.31 |  |
|  |  |  |  |  | Exp Post | 7.34±1.51 | 57.00±16.50 | 8.89±2.46 | 52.02±45.70 | 2.70±3.79 |  |
|  |  |  |  |  | Con Pre | 7.79±1.41 | 62.00±15.40 | 8.34±2.75 | 61.81±34.10 | 2.95±1.55 |  |
|  |  |  |  |  | Con Post | 7.45±1.44 | 58.00±15.70 | 8.88±2.02 | 62.57±28.68 | 3.38±1.69 |  |
| Wong et al., 2009 | Parallel | 12 | 1.60 EPA + 1.00 DHA | 97  (49 vs. 48) | Exp Pre | - | - | 8.10±2.60 | - | - | - |
|  |  |  |  |  | Exp Post |  |  | 7.90±2.20 |  |  |  |
|  |  |  |  |  | Con Pre |  |  | 7.20±1.50 |  |  |  |
|  |  |  |  |  | Con Post |  |  | 6.90±1.30 |  |  |  |
| Westerveld et al., 1993 (Arm 1) | Parallel | 8 | 1.80 EPA | 16  (8 vs. 8) | Exp Pre | 8.20±2.80 | 66.00±30.60 | - | - | - | - |
|  |  |  |  |  | Exp Post | 7.90±2.10 | 63.00±23.00 |  |  |  |  |
| Arm 2 | Parallel | 8 | 0.90 EPA | 16  (8 vs. 8) | Exp Pre | 7.60±2.90 | 60.00±31.70 |  |  |  |  |
|  |  |  |  |  | Exp Post | 8.10±2.80 | 65.00±30.60 |  |  |  |  |
|  |  |  |  |  | Con Pre | 9.20±2.70 | 77.00±29.50 |  |  |  |  |
|  |  |  |  |  | Con Post | 9.30±3.00 | 78.00±32.80 |  |  |  |  |
| Woodman et al., 2002 (Arm 1) | Parallel | 6 | 4.00 EPA | 33 (17 vs. 16) | Exp Pre | 7.14±1.03 | 55.00±11.30 | 7.46±1.81 | 98.34±50.42 | - | 0.95±0.41 |
|  |  |  |  |  | Exp Post | 7.21±1.07 | 55.00±11.70 | 8.49±2.19 | 96.12±49.24 |  | 0.93±0.37 |
| Arm 2 | Parallel | 6 | 4.00 DHA | 34 (18 vs. 16) | Exp Pre | 7.48±0.72 | 58.00±7.90 | 8.25±0.98 | 114.87±62.16 |  | 1.19±0.55 |
|  |  |  |  |  | Exp Post | 7.33±0.85 | 57.00±9.30 | 8.80±1.23 | 110.98±48.27 |  | 1.11±0.38 |
|  |  |  |  |  | Con Pre | 7.14±0.60 | 55.00±6.60 | 7.96±1.60 | 101.19±53.89 |  | 1.14±0.44 |
|  |  |  |  |  | Con Post | 7.04±0.60 | 53.00±6.60 | 7.55±1.36 | 95.08±53.34 |  | 0.99±0.24 |
| Zeman et al., 2006 | Cross Over | 13 | 2.10 EPA + 1.03 DHA | 24 | Exp | 7.53±0.69 | 59.00±7.50 | 9.90±0.70 | - | - | - |
|  |  |  |  |  | Con | 7.56±1.74 | 59.00±19.00 | 9.70±0.80 |  |  |  |
| Zheng et al., 2016 | Parallel | 13 | 1.12 EPA + 0.80 DHA | 113  (58 vs. 55) | Exp Pre | 10.10±4.20 | 87.00±45.90 | 8.18±3.03 | 102.09±60.42 | 5.48±3.97 | - |
|  |  |  |  |  | Exp Post | 8.80±1.90 | 73.00±20.80 | 8.46±3.02 | 80.56±46.94 | 4.68±4.12 |  |
|  |  |  |  |  | Con Pre | 9.20±2.40 | 77.00±26.20 | 8.31±3.63 | 114.59±111.81 | 6.21±5.89 |  |
|  |  |  |  |  | Con Post | 8.80±2.30 | 73.00±25.10 | 8.22±2.51 | 125.70±137.51 | 6.46±6.46 |  |

EPA, eicosapentaenoic acid; DHA, docosahexaenoic acid; HbA1c, glycated haemoglobin; FPG, fasting plasma glucose, HOMA-IR, homeostasis model assessment of insulin resistance; CO, cross over design; WO, wash out period; NS, EPA/DHA not specified; N, number of participants; exp, experimental condition; con, control condition; *, data provided by author; †First 2 months, ‡ remaining 4.

**Table S3.** The effect of n-3 PUFAs on lipid profiles

| **Study** | **Intervention** | | | | **Outcomes** | | | | | | |
| --- | --- | --- | --- | --- | --- | --- | --- | --- | --- | --- | --- |
|  | **Design** | **Duration** | **Dosage** | **N** |  | **TG** | **TC** | **HDL** | **LDL** | **VLDL-C** | **VLDL-TG** |
|  |  | (weeks) | (grams) | (exp vs. con) |  | (mmol.L^-1^) | (mmol.L^-1^) | (mmol.L^-1^) | (mmol.L^-1^) | (mmol.L^-1^) | (mmol.L^-1^) |
| Axelrod et al., 1994 | Parallel | 6 | 1.55 EPA + 1.05g DHA | 18 (9 vs. 9) | Exp Pre | 1.68±1.15 | 5.22±0.78 | - | - | - | - |
|  |  |  |  |  | Exp Pre | 1.25±0.99 | 5.12±1.20 | - | - | - | - |
|  |  |  |  |  | Con Pre | 2.34±0.88 | 5.72±1.17 |  |  |  |  |
|  |  |  |  |  | Con Post | 2.39±1.56 | 4.63±1.04 |  |  |  |  |
| Barbosa et al., 2017* | Parallel | 8 | 1.11 EPA+ 0.69 EPA | 14 (6 vs. 8) | Exp Pre | 1.17±0.55 | 4.57±1.12 | 1.01±0.15 | 3.51±1.10 | - | - |
|  |  |  |  |  | Exp Post | 1.27±0.70 | 3.94±0.85 | 1.05±0.28 | 2.31±0.73 |  |  |
|  |  |  |  |  | Con Pre | 2.58±1.60 | 5.13±1.46 | 0.86±0.15 | 3.30±1.16 |  |  |
|  |  |  |  |  | Con Post | 2.04±0.91 | 4.49±0.82 | 0.92±0.10 | 2.73±0.65 |  |  |
| Farsi et al., 2014 | Parallel | 10 | 1.40 EPA + 0.90 DHA | 44  (22 vs. 22) | Exp Pre | 1.77±0.81 | 5.92±1.55 | 1.18±0.28 | 2.79±0.83 | - | - |
|  |  |  |  |  | Exp Post | 1.49±0.68 | 5.29±1.00 | 1.12±0.28 | 2.71±0.61 |  |  |
|  |  |  |  |  | Con Pre | 1.55±1.13 | 5.76±1.21 | 1.19±0.25 | 2.38±0.73 |  |  |
|  |  |  |  |  | Con Post | 1.65±0.91 | 5.58±1.30 | 1.22±0.29 | 2.54±0.59 |  |  |
| Jacobo-Cejudo et al., 2017 | Parallel | 26 | 0.30 EPA + 0.20 DHA | 54  (29 vs. 25) | Exp Pre | 2.10±0.97 | 5.27±0.87 | 1.13±0.21 | 3.39±0.90 | - | - |
|  |  |  |  |  | Exp Post | 1.55±0.74 | 5.16±1.23 | 1.25±0.38 | 3.36±1.16 |  |  |
|  |  |  |  |  | Con Pre | 3.04±1.91 | 4.67±0.79 | 0.99±0.25 | 2.83±0.89 |  |  |
|  |  |  |  |  | Con Post | 2.84±1.69 | 5.43±0.95 | 1.04±0.24 | 3.30±1.00 |  |  |
| Kabir et al., 2007 | Parallel | 8 | 1.08 EPA + 0.72 DHA | 26 (12 vs. 14) | Exp Pre | 1.20±0.66 | 5.10±0.69 | 1.40±0.35 | 3.50±0.69 | - | - |
|  |  |  |  |  | Exp Post | 1.05±0.59 | 5.20±0.69 | 1.50±0.35 | 3.50±0.69 |  |  |
|  |  |  |  |  | Con Pre | 1.05±0.45 | 5.30±0.75 | 1.40±0.37 | 3.60±0.75 |  |  |
|  |  |  |  |  | Con Post | 1.16±0.49 | 5.20±0.75 | 1.50±0.37 | 3.60±0.75 |  |  |
| Lobraico et al., 2015 | Cross Over | 4 (2 WO) | 1.00 (NS) | 47 | Exp | 1.65±1.10 | 3.94±0.80 | 1.23±0.35 | 1.98±0.48 | - | - |
|  |  |  |  |  | Con | 1.58±1.00 | 3.82±0.79 | 1.20±0.40 | 1.09±0.61 |  |  |
| Lee et al., 2013 (Arm 1) | Parallel | 8 | 0.93 EPA + 0.75 DHA | 34 (17 vs. 17) | Exp Pre | 3.34±0.93 | 4.19±0.62 | 1.00±0.20 | 1.91±0.44 | - | - |
|  |  |  |  |  | Exp Post | 1.94±0.83 | 3.78±0.74 | 1.01±0.25 | 1.91±0.57 |  |  |
| Arm 2 | Parallel | 8 | 1.84 EPA + 1.50 DHA | 32 (15 vs. 17) | Exp Pre | 3.21±0.77 | 4.19±0.54 | 1.09±0.31 | 1.95±0.50 |  |  |
|  |  |  |  |  | Exp Post | 1.97±1.08 | 3.95±0.55 | 1.07±0.28 | 1.86±0.49 |  |  |
|  |  |  |  |  | Con Pre | 3.63±1.57 | 4.42±0.61 | 1.09±0.23 | 2.12±0.78 |  |  |
|  |  |  |  |  | Con Post | 2.76±1.55 | 4.08±0.76 | 1.19±0.35 | 2.00±0.46 |  |  |
| Lee et al., 2014* | Parallel | 8 | 3.58 EPA + 2.44 DHA | 27 (13 vs. 14) | Exp Pre | 1.88±0.88 | 4.60±0.92 | 1.11±0.30 | 2.63±0.95 | 0.86±0.40 |  |
|  |  |  |  |  | Exp Post | 1.73±0.90 | 4.73±1.10 | 1.18±0.31 | 2.75±0.99 | 0.79±0.41 |  |
|  |  |  |  |  | Con Pre | 1.96±1.31 | 4.65±1.05 | 1.22±0.36 | 2.67±0.99 | 0.76±0.31 |  |
|  |  |  |  |  | Con Post | 2.01±1.07 | 4.62±1.16 | 1.24±0.39 | 2.49±1.03 | 0.92±0.49 |  |
| Luo et al., 1998 | Cross Over | 8 (8 WO) | 1.08 EPA + 0.72 DHA | 10 | Exp | 1.73±0.85 | 5.69±1.04 | 1.10±0.16 | 2.59±0.66 | - | - |
|  |  |  |  |  | Con | 2.21±1.11 | 5.72±1.26 | 1.03±0.16 | 2.49±0.73 |  |  |
| Mahmoudabadi et al., 2014 | Parallel | 8 | 0.50 EPA | 41 (21 vs. 20) | Exp Pre | 2.30±0.60 | - | - | - | - | - |
|  |  |  |  |  | Exp Post | 1.94±0.51 |  |  |  |  |  |
|  |  |  |  |  | Con Pre | 1.52±0.81 |  |  |  |  |  |
|  |  |  |  |  | Con Post | 1.26±0.83 |  |  |  |  |  |
| Mansoori et al., 2015 | Parallel | 8 | 0.40 EPA + 1.45 DHA | 68 (35 vs. 33) | Exp Pre | 2.18±1.22 | 4.80±1.21 | 1.21±0.24 | 2.50±0.72 | - | - |
|  |  |  |  |  | Exp Post | 1.62±0.72 | 4.76±1.19 | 1.15±0.32 | 2.58±0.76 |  |  |
|  |  |  |  |  | Con Pre | 1.79±0.79 | 4.51±0.71 | 1.22±0.23 | 2.31±0.45 |  |  |
|  |  |  |  |  | Con Post | 1.97±0.99 | 4.36±0.80 | 1.10±0.21 | 2.34±0.55 |  |  |
| McGrath et al., 1996 | Cross Over | 6 (6 WO) | 1.80 EPA + 1.60 DHA | 23 | Exp | 1.40±0.81 | 5.30±0.92 | 1.14±0.50 | 3.78±1.49 | 0.46±0.50 | 1.18±1.25 |
|  |  |  |  |  | Con | 1.60±0.58 | 5.30±0.92 | 1.09±0.43 | 3.53±1.41 | 0.62±0.49 | 1.32±0.94 |
| Mita et al., 2007 | Parallel | 104 | 1.80 EPA | 60 (30 vs. 30) | Exp Pre | 1.59±1.11 | 5.37±0.74 | 1.40±0.44 | - | - | - |
|  |  |  |  |  | Exp Post | 1.77±1.07 | 5.15±0.83 | 1.51±0.59 |  |  |  |
|  |  |  |  |  | Con Pre | 1.51±0.63 | 5.37±1.03 | 1.45±0.40 |  |  |  |
|  |  |  |  |  | Con Post | 1.51±0.90 | 5.27±0.99 | 1.44±0.37 |  |  |  |
| Morgan et al., 1995 | Parallel | 12 | 2.59-5.18 EPA + 2.46-4.91 DHA | 40 (20 vs. 20) | Exp Pre | 6.13±3.12 | 6.35±0.87 | 1.05±0.37 | 3.71±0.78 | 1.01±0.68 | 4.16±2.76 |
|  |  |  |  |  | Exp Post | 4.67±2.61 | 6.49±0.98 | 1.00±0.45 | 4.08±0.78 | 0.71±0.56 | 2.63±1.87 |
|  |  |  |  |  | Con Pre | 7.80±4.83 | 6.51±1.28 | 0.94±0.26 | 3.87±1.30 | 1.06±0.66 | 4.75±3.34 |
|  |  |  |  |  | Con Post | 8.59±7.53 | 6.83±1.05 | 0.93±0.27 | 3.87±1.43 | 1.28±1.15 | 5.82±6.19 |
| Mostad et al., 2006 | Parallel | 8 | 1.80 EPA + 3.00 DHA | 26 (12 vs. 14) | Exp Pre | 1.60±0.79 | 5.00±1.10 | 1.27±0.29 | 3.00±0.86 | - | - |
|  |  |  |  |  | Exp Post | 1.60±0.71 | 5.00±0.94 | 1.32±0.41 | 3.00±0.94 |  |  |
|  |  |  |  |  | Con Pre | 1.50±0.69 | 5.00±1.04 | 1.15±0.24 | 3.20±0.87 |  |  |
|  |  |  |  |  | Con Post | 1.40±0.61 | 4.70±0.95 | 1.20±0.28 | 2.90±0.87 |  |  |
| Neil et al., 2010 | Parallel | 17 | 1.68 EPA + 0.92 DHA | 326  (160 vs. 166) | Exp Pre | 1.60±0.89 | 5.00±0.80 | 1.2±0.03 | 3.10±0.70 | 0.70±0.30 | - |
|  |  |  |  |  | Exp Post | 1.40±0.89 | 5.00±0.90 | 1.2±0.03 | 3.10±0.70 | 0.70±0.40 |  |
|  |  |  |  |  | Con Pre | 1.50±0.74 | 5.00±1.00 | 1.2±0.03 | 3.20±0.80 | 0.70±0.30 |  |
|  |  |  |  |  | Con Post | 1.50±0.74 | 5.00±1.00 | 1.2±0.03 | 3.10±0.80 | 0.70±0.30 |  |
| Peliknov et al., 1993 | Parallel | 4 | 3.10 (NS) | 20  (10 vs. 10) | Exp Pre | 1.53±0.65 | 6.44±1.20 | - | - | - | - |
|  |  |  |  |  | Exp Post | 1.49±0.97 | 6.62±0.88 |  |  |  |  |
|  |  |  |  |  | Con Pre | 2.01±0.60 | 6.22±0.72 |  |  |  |  |
|  |  |  |  |  | Con Post | 1.90±0.70 | 6.13±1.00 |  |  |  |  |
| Petersen et al., 2002 | Parallel | 8 | 2.60 (NS) | 42 (20 vs. 22) | Exp Pre | 2.35±1.21 | 5.95±0.94 | 1.22±0.22 | 3.29±0.67 | - | - |
|  |  |  |  |  | Exp Post | 1.81±0.89 | 5.97±1.03 | 1.28±0.27 | 3.43±0.76 |  |  |
|  |  |  |  |  | Con Pre | 2.76±2.16 | 5.51±1.08 | 1.12±0.23 | 2.79±0.75 |  |  |
|  |  |  |  |  | Con Post | 2.71±2.30 | 5.43±0.94 | 1.11±0.28 | 2.87±0.48 |  |  |
| Rivellese et al., 1996 | Parallel | 26 | 0.96 EPA + 1.59 DHA | 16 (8 vs. 8) | Exp Pre | 3.85±0.91 | 6.26±1.61 | 0.88±0.11 | 2.88±0.57 | 2.77±2.15 | 4.25±1.87 |
|  |  |  |  |  | Exp Post | 2.92±0.65 | 5.71±0.74 | 0.89±0.14 | 3.29±1.39 | 1.47±0.37 | 2.35±0.68 |
|  |  |  |  |  | Con Pre | 3.29±1.07 | 5.76±0.82 | 0.93±0.20 | 3.38±0.57 | 1.59±0.57 | 2.76±1.13 |
|  |  |  |  |  | Con Post | 3.85±0.91 | 6.27±1.61 | 0.95±0.28 | 3.30±1.07 | 1.99±2.50 | 2.97±2.18 |
| Sasaki et al., 2012 | Parallel | 26 | 1.80 EPA | 28 (15 vs. 13) | Exp Pre | 1.76±1.36 | 4.71±0.88 | 1.45±0.36 | 2.49±0.62 | - | - |
|  |  |  |  |  | Exp Post | 1.84±1.18 | 4.43±0.93 | 1.42±0.41 | 2.18±0.75 |  |  |
|  |  |  |  |  | Con Pre | 1.39±0.50 | 4.71±0.60 | 1.55±0.33 | 2.49±0.52 |  |  |
|  |  |  |  |  | Con Post | 1.29±0.52 | 4.79±0.67 | 1.50±0.34 | 2.62±0.52 |  |  |
| Schectman et al., 1988 | CO | 4 (4 WO) | 2.60 EPA + 1.40 DHA | 13 | Exp | 1.71±0.77 | 5.75±1.12 | 0.76±0.23 | 3.68±1.21 | 0.65±0.47 | 1.07±0.73 |
|  |  |  |  |  | Con | 2.27±1.02 | 5.21±0.84 | 0.81±0.17 | 3.37±0.75 | 0.67±0.47 | 1.48±0.98 |
| Shidfar et al., 2008 | Parallel |  | 0.52 EPA + 0.48 DHA | 50 (25 vs. 25) | Exp Pre | 3.39±0.32 | 6.33±0.80 | 1.01±0.23 | 4.16±1.07 | - | - |
|  |  |  |  |  | Exp Post | 2.32±0.27 | 6.07±0.99 | 0.96±0.26 | 4.09±1.14 |  |  |
|  |  |  |  |  | Con Pre | 2.33±0.44 | 6.46±1.17 | 0.99±0.21 | 4.36±0.96 |  |  |
|  |  |  |  |  | Con Post | 3.64±0.41 | 6.16±0.90 | 0.98±0.24 | 4.41±1.07 |  |  |
| Udupa et al., 2012 | Parallel | 13 | 1.08 EPA + 0.72 DHA | 29 (16 vs. 13) | Exp Pre | - | 5.67±0.45 | - | - | - | - |
|  |  |  |  |  | Exp Post |  | 5.14±0.53 |  |  |  |  |
|  |  |  |  |  | Con Pre |  | 5.77±0.69 |  |  |  |  |
|  |  |  |  |  | Con Post |  | 5.47±0.63 |  |  |  |  |
| Vanschoonbeek et al., 2007 | Parallel | 4 | 2.03 EPA + 1.13 DHA | 42 (20 vs. 22) | Exp Pre | 2.36±1.21 | 5.96±0.94 | 1.22±0.22 | 3.29±0.67 | - | - |
|  |  |  |  |  | Exp Post | 1.87±1.17 | 5.89±0.92 | 1.29±0.23 | 3.45±0.69 |  |  |
|  |  |  |  |  | Con Pre | 2.76±2.16 | 5.51±1.08 | 1.12±0.23 | 2.79±0.75 |  |  |
|  |  |  |  |  | Con Post | 2.70±2.05 | 5.47±1.06 | 1.11±0.23 | 2.91±0.77 |  |  |
| Veleba et al., 2015 | Parallel | 24 | 0.75 EPA + 2.00 DHA | 29 (16 vs. 13) | Exp Pre | 2.14±1.02 | 4.86±0.71 | 1.13±0.27 | 2.66±0.58 | - | - |
|  |  |  |  |  | Exp Post | 1.83±0.63 | 4.72±0.58 | 1.07±0.27 | 2.87±0.55 |  |  |
|  |  |  |  |  | Con Pre | 1.48±0.44 | 4.74±1.33 | 1.13±0.24 | 2.58±1.15 |  |  |
|  |  |  |  |  | Con Post | 1.60±0.98 | 4.43±1.48 | 1.00±0.32 | 2.51±0.93 |  |  |
| Véricel et al., 2015 | CO | 2 (6 WO) | 0.40 DHA | 11 | Exp | 1.51±0.53 | 4.47±1.09 | 1.18±0.27 | 2.53±1.00 | - | - |
|  |  |  |  |  | Con | 1.91±0.81 | 4.30±0.83 | 1.12±0.23 | 2.35±0.70 |  |  |
| Wang et al., 2016 | Parallel | 26 | 1.34 EPA + 1.07 DHA | 99  (49 vs. 50) | Exp Pre | 1.60±0.92 | 4.63±0.76 | 1.36±0.35 | 3.34±0.70 | - | - |
|  |  |  |  |  | Exp Post | 1.26±0.66 | 5.28±0.80 | 1.56±0.39 | 4.11±0.95 |  |  |
|  |  |  |  |  | Con Pre | 1.73±0.90 | 4.44±0.94 | 1.26±0.27 | 3.27±0.85 |  |  |
|  |  |  |  |  | Con Post | 1.78±0.95 | 5.19±1.08 | 1.35±0.28 | 4.08±1.07 |  |  |
| Wong et al., 2009 | Parallel | 12 | 1.60 EPA + 1.00 DHA | 97  (49 vs. 48) | Exp Pre | 1.50±1.10 | 5.00±0.90 | 1.40±0.36 | 3.00±0.80 | - | - |
|  |  |  |  |  | Exp Post | 1.20±0.50 | 4.50±0.90 | 1.29±0.37 | 2.80±0.80 |  |  |
|  |  |  |  |  | Con Pre | 1.40±0.80 | 4.80±1.00 | 1.32±0.27 | 2.80±0.70 |  |  |
|  |  |  |  |  | Con Post | 1.60±1.00 | 4.60±0.80 | 1.20±0.25 | 2.70±0.70 |  |  |
| Woodman et al., 2002 (Arm 1) | Parallel | 6 | 4.00 EPA | 33  (17 vs. 16) | Exp Pre | 1.34±0.74 | 4.48±1.03 | 1.21±0.37 | 2.66±0.82 | - | - |
|  |  |  |  |  | Exp Post | 1.11±0.58 | 4.42±0.87 | 1.22±0.37 | 2.68±0.78 |  |  |
| Arm 2 | Parallel | 6 | 4.00 DHA | 34  (18 vs. 16) | Exp Pre | 1.62±0.59 | 4.49±0.64 | 0.99±0.21 | 2.74±0.55 | - | - |
|  |  |  |  |  | Exp Post | 1.35±0.55 | 4.55±0.76 | 1.03±0.21 | 2.88±0.59 |  |  |
|  |  |  |  |  | Con Pre | 1.74±0.60 | 4.57±0.68 | 1.06±0.24 | 2.71±0.48 |  |  |
|  |  |  |  |  | Con Post | 1.68±0.56 | 4.61±0.68 | 1.07±0.24 | 2.77±0.44 |  |  |
| Zeman et al., 2006 | Cross Over | 13 | 2.10 EPA + 1.03 DHA | 24 | Exp | 3.45±0.67 | 5.69±0.39 | - | - | - | - |
|  |  |  |  |  | Con | 4.14±0.92 | 5.90±0.45 |  |  |  |  |
| Zheng et al., 2016 | Parallel | 13 | 1.12 EPA + 0.80 DHA | 113  (58 vs. 55) | Exp Pre | 1.68±0.74 | 4.66±0.96 | 1.14±0.30 | 2.97±0.80 | - | - |
|  |  |  |  |  | Exp Post | 1.45±0.75 | 4.50±1.00 | 1.23±0.30 | 2.62±0.81 |  |  |
|  |  |  |  |  | Con Pre | 1.85±0.97 | 4.88±1.01 | 1.19±0.20 | 3.05±0.85 |  |  |
|  |  |  |  |  | Con Post | 1.75±0.87 | 5.04±1.10 | 1.25±0.25 | 2.99±0.90 |  |  |

EPA, eicosapentaenoic acid; DHA, docosahexaenoic acid; TG, triglycerides; TC, total cholesterol; HDL, high- density lipoprotein cholesterol; LDL, low-density lipoprotein cholesterol; VLDL-C, very low-density lipoprotein cholesterol; VLDL-TG, very low-density lipoprotein triglycerides; WO, wash out period; NS, EPA/DHA not specified; exp, experimental condition; con, control condition; *****, data provided by author; **~,** varied units.

**Table S4.** The effect of n-3 PUFAs on Inflammatory parameters, and blood pressure.

| **Study** | **Intervention** | | | | **Outcomes** | | | | | |
| --- | --- | --- | --- | --- | --- | --- | --- | --- | --- | --- |
|  | **Design** | **Duration** | **Dosage** | **N** |  | **CRP** | **TNF-α** | **IL-6** | **SBP** | **DBP** |
|  |  | (weeks) | (g/day) | (exp vs con) |  | (nmol.L^-1^) | (pg.mL^-1^) | (pg.mL^-1^) | (mmHg) | (mmHg) |
| Ansari et al., 2017 | Parallel | 10 | 1.80 EPA + 0.90 DHA | 43 (22 vs. 21) | Exp Pre | - | - | - | 127.20±8.91 | 81.66±7.69 |
|  |  |  |  |  | Exp Post |  |  |  | 127.10±7.97 | 81.45±5.58 |
|  |  |  |  |  | Con Pre |  |  |  | 131.70±10.08 | 85.20±9.11 |
|  |  |  |  |  | Con Post |  |  |  | 130.20±10.08 | 86.00±8.52 |
| Axelrod et al., 1994 | Parallel | 6 | 1.55 EPA + 1.05g DHA | 18 (9 vs. 9) | Exp Pre | - | - | - | 128.90±18.90 | - |
|  |  |  |  |  | Exp Post |  |  |  | 125.25±24.81 |  |
|  |  |  |  |  | Con Pre |  |  |  | 132.40±22.80 |  |
|  |  |  |  |  | Con Post |  |  |  | 143.89±36.95 |  |
| Azizi-Soleiman et al., 2002 (Arm 1) | Parallel | 12 | 0.98 EPA | 31 (14 vs. 17) | Exp Pre | 20961.90±25323.81 | - | - | - | - |
|  |  |  |  |  | Exp Post | 25952.38±32838.10 |  |  |  |  |
| (Arm 2) | Parallel | 12 | 0.98 DHA | 31 (14 vs. 17) | Exp Pre | 27133.33±34171.43 |  |  |  |  |
|  |  |  |  |  | Exp Post | 20800.00±28371.43 |  |  |  |  |
|  |  |  |  |  | Con Pre | 20457.14±21838.10 |  |  |  |  |
|  |  |  |  |  | Con Post | 23038.10±23704.76 |  |  |  |  |
| Barbosa et al., 2017* | Parallel | 8 | 1.11 EPA+ 0.69 EPA | 14 (6 vs. 8) | Exp Pre | - | - | - | 138.33±25.95 | 81.33±16.48 |
|  |  |  |  |  | Exp Post |  |  |  | 130.00±18.04 | 75.17±9.39 |
|  |  |  |  |  | Con Pre |  |  |  | 133.50±26.53 | 80.88±14.57 |
|  |  |  |  |  | Con Post |  |  |  | 125.88±12.56 | 76.38±5.66 |
| Balfegó et al., 2016 | Parallel | 26 | 3.00 ± 0.20 (NS) | 32 (17 vs. 15) | Exp Pre | 19.05±15.24 | 5.60±0.87 | 3.50±1.30 | - | - |
|  |  |  |  |  | Exp Post | 19.05±14.23 | 5.60±1.65 | 3.60±1.65 |  |  |
|  |  |  |  |  | Con Pre | 19.05±41.90 | 4.90±1.20 | 3.00±1.20 |  |  |
|  |  |  |  |  | Con Post | 19.05±38.10 | 4.90±1.20 | 3.30±1.54 |  |  |
| Holman et al., 2008 | Parallel | 17 | 1.68 EPA + 0.92 DHA | 361 (183 vs. 178) | Exp Pre | - | - | - | 135.70±15.50 | 76.90±8.90 |
|  |  |  |  |  | Exp Post |  |  |  | 138.00±15.67 | 79.00±8.98 |
|  |  |  |  |  | Con Pre |  |  |  | 139.80±15.90 | 78.80±9.20 |
|  |  |  |  |  | Con Post |  |  |  | 141.45±16.08 | 80.23±9.30 |
| Jazayery et al., 2012 | Parallel | 8 | 0.50 EPA | 33  (16 vs. 17) | Exp Pre | - | 11.26±1.10 | 16.90±0.54 | - | - |
|  |  |  |  |  | Exp Post |  | 10.05±0.54 | 14.03±0.30 |  |  |
|  |  |  |  |  | Con Pre |  | 12.80±1.04 | 17.34±0.60 |  |  |
|  |  |  |  |  | Con Post |  | 13.53±0.74 | 18.94±0.17 |  |  |
| Kabir et al., 2007 | Parallel | 8 | 1.08 EPA + 0.72 DHA | 26 (12 vs. 14) | Exp Pre | - | 5.50±13.15 | 2.80±1.73 | - | - |
|  |  |  |  |  | Exp Post |  | 6.30±17.30 | 3.10±2.08 |  |  |
|  |  |  |  |  | Con Pre |  | 7.20±21.31 | 1.30±1.87 |  |  |
|  |  |  |  |  | Con Post |  | 7.50±22.81 | 2.40±2.12 |  |  |
| Lee et al., 2014* | Parallel | 8 | 3.58 EPA + 2.44 DHA | 27  (13 vs. 14) | Exp Pre | 69.90±132.67 | - | - | 127.77±15.43 | 71.15±9.41 |
|  |  |  |  |  | Exp Post | 50.00±69.52 |  |  | 130.23±19.08 | 69.31±9.67 |
|  |  |  |  |  | Con Pre | 23.24±22.10 |  |  | 132.29±22.15 | 73.71±13.10 |
|  |  |  |  |  | Con Post | 22.57±32.76 |  |  | 131.57±13.75 | 71.28±12.81 |
| McVeigh et al., 1994 | Cross Over | 6 (6 WO) | 1.80 EPA + 1.20 DHA | 23 | Exp | - | - | - | 103.00±14.96 | 68.00±6.41 |
|  |  |  |  |  | Con |  |  |  | 107.00±18.16 | 70.00±8.55 |
| Mita et al., 2007 | Parallel | 104 | 1.80 EPA | 60  (30 vs. 30) | Exp Pre | - | - | - | 134.30±19.60 | 78.90±11.30 |
|  |  |  |  |  | Exp Post |  |  |  | 129.80±15.50 | 76.30±9.20 |
|  |  |  |  |  | Con Pre |  |  |  | 141.50±18.90 | 82.60±10.40 |
|  |  |  |  |  | Con Post |  |  |  | 138.30±17.60 | 81.20±12.10 |
| Moghadam et al., 2012 | Parallel | 8 | 1.55 EPA + 0.83 DHA | 84 (42 vs. 42) | Exp Pre | 244.57±260.76 | 37.52±6.41 | - | - | - |
|  |  |  |  |  | Exp Post | 193.81±230.38 | 34.46±6.40 |  |  |  |
|  |  |  |  |  | Con Pre | 177.81±159.52 | 38.68±9.53 |  |  |  |
|  |  |  |  |  | Con Post | 173.05±107.90 | 40.67±11.01 |  |  |  |
| Mori et al., 2003 (Arm 1) | Parallel | 6 | 3.84 EPA | 33 (17 vs. 16) | Exp Pre | 21.71±21.62 | 24.44±13.78 | 1.75±0.78 | - | - |
|  |  |  |  |  | Exp Post | 19.14±16.86 | 19.67±11.57 | 1.78±0.51 |  |  |
| (Arm 2) | Parallel | 6 | 3.68 DHA | 33 (17 vs. 16) | Exp Pre | 35.24±24.00 | 20.51±13.46 | 2.22±1.87 |  |  |
|  |  |  |  |  | Exp Post | 36.57±28.67 | 13.78±10.20 | 2.15±1.47 |  |  |
|  |  |  |  |  | Con Pre | 19.14±21.71 | 15.35±10.62 | 1.76±0.79 |  |  |
|  |  |  |  |  | Con Post | 20.19±21.71 | 14.26±7.73 | 1.96±0.83 |  |  |
| Pooya et al., 2010 | Parallel | 8 | 1.55 EPA + 0.83 DHA | 81  (40 vs. 41) | Exp Pre | 25.71±0.19 | - | - | - | - |
|  |  |  |  |  | Exp Post | 23.62±2.19 |  |  |  |  |
|  |  |  |  |  | Con Pre | 30.00±3.43 |  |  |  |  |
|  |  |  |  |  | Con Post | 36.19±1.62 |  |  |  |  |
| Sasaki et al., 2012 | Parallel | 26 | 1.80 EPA | 28  (15 vs. 13) | Exp Pre | - | - | - | 129.00±11.00 | 67.00±10.00 |
|  |  |  |  |  | Exp Post |  |  |  | 130.00±15.00 | 69.00±12.00 |
|  |  |  |  |  | Con Pre |  |  |  | 127.00±14.00 | 69.00±9.00 |
|  |  |  |  |  | Con Post |  |  |  | 128.00±15.00 | 74.00±10.00 |
| Veleba et al., 2015 | Parallel | 24 | 0.75 EPA + 2.0 DHA | 29 (16 vs. 13) | Exp Pre | - | - | - | 144.67±14.53 | 88.33±7.26 |
|  |  |  |  |  | Exp Post |  |  |  | 139.33±15.33 | 83.67±12.11 |
|  |  |  |  |  | Con Pre |  |  |  | 138.67±16.58 | 84.00±13.27 |
|  |  |  |  |  | Con Post |  |  |  | 139.00±12.44 | 81.67±16.58 |
| Véricel et al., 2015 | CO | 2 (6 WO) | 0.40 DHA | 11 | Exp | - | - | - | 141.20±17.25 | 77.80±9.95 |
|  |  |  |  |  | Con |  |  |  | 137.30±20.90 | 74.20±9.29 |
| Wong et al., 2009 | Parallel | 12 | 1.60 EPA + 1.00 DHA | 97  (49 vs. 48) | Exp Pre | 12.95±13.52 | - | - | - | - |
|  |  |  |  |  | Exp Post | 16.29±29.62 |  |  |  |  |
|  |  |  |  |  | Con Pre | 16.00±24.10 |  |  |  |  |
|  |  |  |  |  | Con Post | 12.19±14.86 |  |  |  |  |
| Woodman et al., 2002 (Arm 1) | Parallel | 6 | 4.00 EPA | 33 (17 vs. 16) | Exp Pre | - | - | - | 137.10±16.90 | 75.80±9.07 |
|  |  |  |  |  | Exp Post |  |  |  | 133.70±14.02 | 74.60±1.90 |
| Arm 2 | Parallel | 6 | 4.00 DHA | 34 (18 vs. 16) | Exp Pre | - | - | - | 138.50±16.54 | 71.80±10.18 |
|  |  |  |  |  | Exp Post |  |  |  | 142.70±4.80 | 71.90±7.63 |
|  |  |  |  |  | Con Pre |  |  |  | 135.90±14.40 | 73.00±6.00 |
|  |  |  |  |  | Con Post |  |  |  | 132.50±11.20 | 72.10±5.20 |

EPA, eicosapentaenoic acid; DHA, docosahexaenoic acid; CRP, C-reactive protein; TNFα, tumor necrosis factor alpha; IL-6, Interleukin 6; SBP, systolic blood pressure; DBP, diastolic blood pressure; cross over design; WO, wash out period; NS, EPA/DHA not specified; exp, experimental condition; con, control condition; *****, data provided by author.

**Table S5.** The effect of n-3 PUFAs on apolipoproteins and non-esterified fatty acids.

| **Study** | **Intervention** |  |  |  | **Outcomes** |  |  |  |
| --- | --- | --- | --- | --- | --- | --- | --- | --- |
|  | **Design** | **Duration** | **Dosage** | **N** |  | **APO-A** | **APO-B** | **NEFA** |
|  |  |  |  | (exp vs con) |  | (g/L^-1^) | (g/L^-1^) | (mmol/L^-1^) |
| Barbosa et al., 2017***** | Parallel | 8 | 1.11 EPA + 0.69 EPA | 14 (6 vs. 8) | Exp Pre | 1.21±0.20 | 0.86±0.24 | 0.61±0.20 |
|  |  |  |  |  | Exp Post | 1.22±0.22 | 0.91±0.18 | 0.45±0.21 |
|  |  |  |  |  | Con Pre | 1.17±0.26 | 1.02±0.34 | 0.58±0.24 |
|  |  |  |  |  | Con Post | 1.26±0.23 | 1.05±0.32 | 0.65±0.23 |
| Farsi et al., 2014 | Parallel | 10 | 1.40 of EPA + 0.90g of DHA | 44 (22 vs. 22) | Exp Pre | **-** | **-** | 1936.73±90.91 (~) |
|  |  |  |  |  | Exp Post |  |  | 1737.72±51.01 |
|  |  |  |  |  | Con Pre |  |  | 1929.64±87.43 |
|  |  |  |  |  | Con Post |  |  | 1997.00±77.17 |
| Lee et al., 2013 (Arm 1) | Parallel | 8 | 0.93 EPA + 0.75 DHA | 34  (17 vs. 17) | Exp Pre | 149.90±19.30 | 82.50±13.80 | - |
|  |  |  |  |  | Exp Post | 155.40±18.40 | 77.10±19.10 |  |
| (Arm 2) | Parallel | 8 | 1.84 EPA + 1.50 DHA | 32  (15 vs. 17) | Exp Pre | 142.00±19.90 | 82.20±16.90 |  |
|  |  |  |  |  | Exp Post | 134.00±21.90 | 80.10±24.10 |  |
|  |  |  |  |  | Con Pre | 144.10±29.40 | 87.50±15.70 |  |
|  |  |  |  |  | Con Post | 142.40±26.30 | 83.20±19.50 |  |
| Luo et al., 1998 | Cross Over | 8 (8 WO) | 1.08 EPA + 0.72 DHA | 10 | Exp | 1.43±0.22 |  | - |
|  |  |  |  |  | Con | 1.48±0.25 |  |  |
| McGrath et al., 1996 | Cross Over | 6 (6 WO) | 1.80 EPA+1.60 DHA | 23 | Exp | 1.12±0.51 | 0.95±0.54 | - |
|  |  |  |  |  | Con | 1.10±0.51 | 0.95±0.51 |  |
| Schectman et al., 1988 | Cross Over | 4 (4 WO) | 2.60 EPA + 1.40 DHA | 13 | Exp | 1.11±0.25 | 1.16±0.29 | - |
|  |  |  |  |  | Con | 1.21±0.43 | 1.09±0.32 |  |
| Shidfar et al., 2008 | Parallel | 10 | 0.52 EPA + 0.48 DHA | 50 (25 vs. 25) | Exp Pre | 1.76±0.49 | - | - |
|  |  |  |  |  | Exp Post | 1.76±0.40 |  |  |
|  |  |  |  |  | Con Pre | 1.74±0.24 |  |  |
|  |  |  |  |  | Con Post | 1.67±0.21 |  |  |
| Veleba et al., 2015 | Parallel | 24 | 0.75 EPA + 2.00 DHA | 29 (16 vs. 13) | Exp Pre | **-** | **-** | 0.70±0.30 |
|  |  |  |  |  | Exp Post |  |  | 0.66±0.25 |
|  |  |  |  |  | Con Pre |  |  | 0.72±0.12 |
|  |  |  |  |  | Con Post |  |  | 0.68±0.30 |
| Zeman et al., 2006 | Cross Over | 13 | 2.10 EPA + 1.03 DHA | 24 | Exp | 1.70±0.07 | **-** | 0.88±0.07 |
|  |  |  |  |  | Con | 1.72±0.07 |  | 0.87±0.11 |

EPA, eicosapentaenoic acid; DHA, docosahexaenoic acid; NEFA, non-esterified fatty acids; Apo-A, apolipoprotein A1; Apo-B, apolipoprotein B; WO, wash out period; ~, expressed as ng/mL^-1^; exp, experimental condition; con, control condition; *****, data provided by author.

**Table S6.** Inclusion/exclusion criteria

| **Criteria** | **Inclusion** | **Exclusion** |
| --- | --- | --- |
| Study type | Randomised controlled trials | Observational studies, reviews, book chapters, case studies, cross over trials that have provided no information on washout |
| Study population | Adults with type 2 diabetes | Participants with any other significant medical condition except type one or two diabetes (e.g. respiratory, cardiovascular, metabolic and psychiatric disease). Gestational diabetes, diabetes insipidus, pre-diabetes (Fasting plasma glucose (mmol/L): 6.0 - 6.9; HbA1c (%): 6.0 - 6.4) |
| Intervention | Omega-3 fatty acid interventions (eicosapentaenoic acid, docosahexaenoic acid, or alpha- linolenic acid) in diet or capsule form where the dosage and duration can be determined | Studies which fail to report the method of supplementation or the dosage. When studies have assessed the effects of omega-3 fatty acids in conjunction with other nutrients or interventions data will only be extracted from the omega-3 only group |
| Outcomes | Include one or more of the following outcomes:  HbA1c, fasting plasma glucose, fasting insulin, homeostatic model of insulin resistance, c-peptide, triglycerides, total cholesterol, high density lipoprotein, low density lipoprotein, very low density lipoprotein cholesterol, very low density lipoprotein triglycerides, apolipoprotein-a, apolipoprotein-b, non-esterified fatty acids, c-reactive protein, tumour necrosis factor alpha, interleukin 6, systolic blood pressure, diastolic blood pressure | No relevant outcome reported |
| Other |  | Animal studies  In vitro studies |

Although studies including people with type 1 diabetes were included in the initial search there was insufficient studies to include them in the meta-analysis, owing to inadequate experimental designs.

**Table S7.** Primary outcome of included studies

| Study | Primary Outcome |
| --- | --- |
| Ansari et al., 2017 | Serum irisin |
| Axelrod et al., 1994 | No primary outcome variable stated |
| Azizi-soleiman et al., 2002 | No primary outcome variable stated |
| Balfego et al., 2016 | No primary outcome variable stated |
| Barbosa et al., 2017 | No primary outcome variable stated |
| Farsi et al., 2014 | No primary outcome variable stated |
| Hosseinzadeh-Attar et al. 2012 | Serum visfatin |
| Jacobo-Cejudo et al., 2017 | No primary outcome variable stated |
| Jazayery et al., 2012 | No primary outcome variable stated |
| Kabir et al., 2007 | No primary outcome variable stated |
| Lee et al., 2013 | Low density lipoprotein particle size |
| Lee et al., 2014 | No primary outcome variable stated |
| Lobraico et al. 2015 | Endothelial function |
| Lou et al., 1998 | No primary outcome variable stated |
| Mahmoudabadi et al. 2014 | No primary outcome variable stated |
| Mansoori et al., 2015 | No primary outcome variable stated |
| Mita et al., 2007 | No primary outcome variable stated |
| McDonald et al., 2012 | No primary outcome variable stated |
| McGrath et al., 1996 | No primary outcome variable stated |
| McVeigh et al., 1994 | No primary outcome variable stated |
| Moghadam et al., 2012 | No primary outcome variable stated |
| Morgan et al., 1995 | No primary outcome variable stated |
| Mori et al., 2003 | No primary outcome variable stated |
| Mostad et al., 2006 | No primary outcome variable stated |
| Neil et al., 2010 | No primary outcome variable stated |
| Pelikinov et al.,1993 | No primary outcome variable stated |
| Petersen et al., 2002 | No primary outcome variable stated |
| Pooya et al., 2010 | No primary outcome variable stated |
| Rivellese et al., 1996 | No primary outcome variable stated |
| Sarbolouki et al., 2013 | No primary outcome variable stated |
| Sasaki et al., 2012 | Endothelium-dependent vasodilation |
| Schectman et al., 1988 | No primary outcome variable stated |
| Shidfar et al., 2008 | No primary outcome variable stated |
| Sitori et al., 1997 | No primary outcome variable stated |
| Toorang et al., 2016 | No primary outcome variable stated |
| Udupa et al., 2012 | No primary outcome variable stated |
| Vanschoonbeek et al., 2007 | No primary outcome variable stated |
| Veleba et al., 2015 | Hyperinsulinemic-euglycemic clamp |
| Véricel et al., 2015 | No primary outcome variable stated |
| Wang et al., 2016 | No primary outcome variable stated |
| Westerveld et al., 1993 | No primary outcome variable stated |
| Wong et al., 2009 | Endothelium-dependent vasodilation |
| Woodman et al., 2002 | No primary outcome variable stated |
| Zeman et al., 2006 | No primary outcome variable stated |
| Zheng et al., 2016 | No primary outcome variable stated |


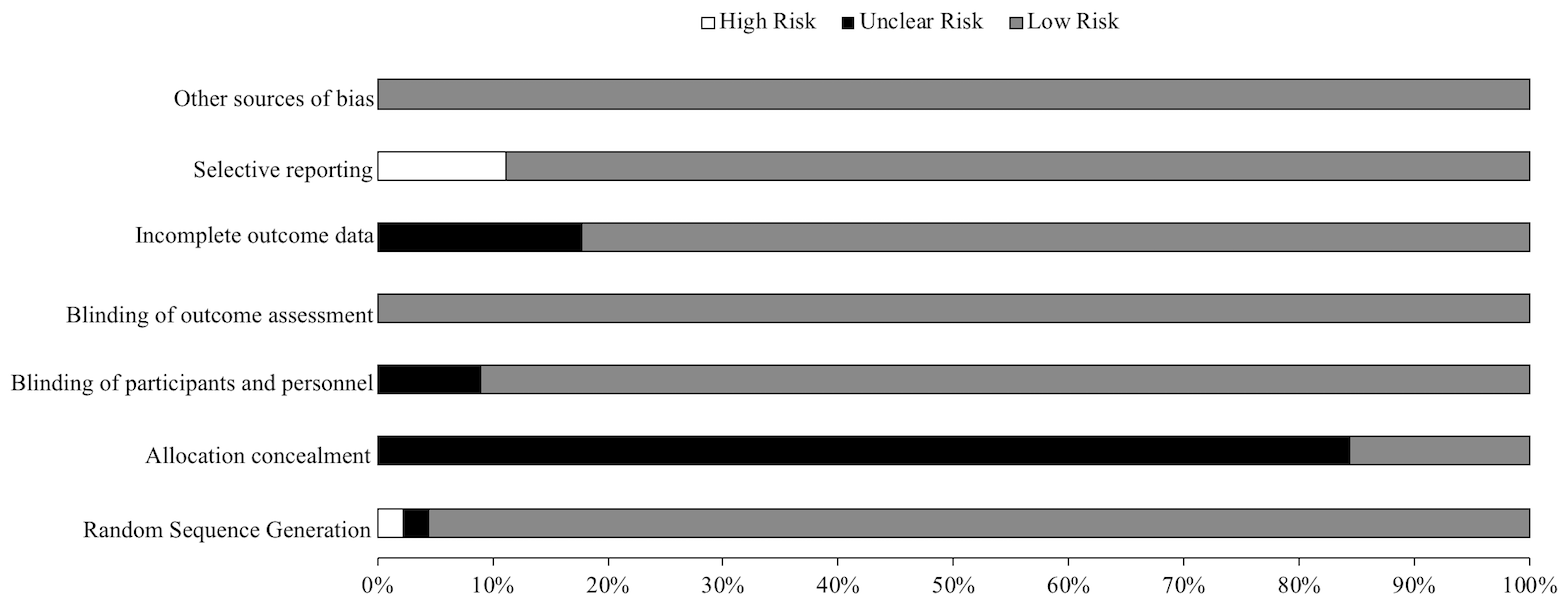


**Figure S1.** Risk of bias across expressed as a percentage across all included studies


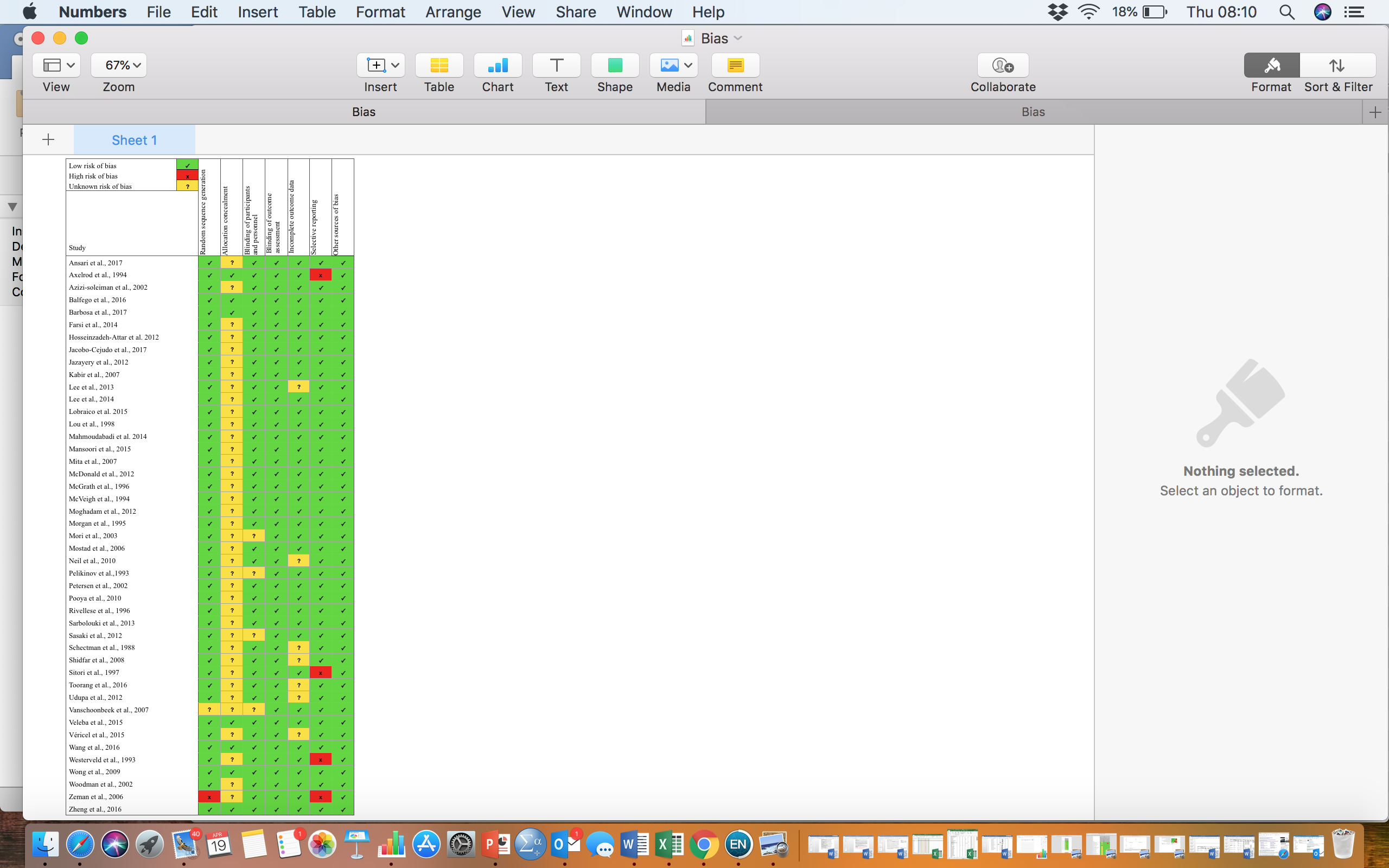


**Figure S2**. Risk of bias figure.


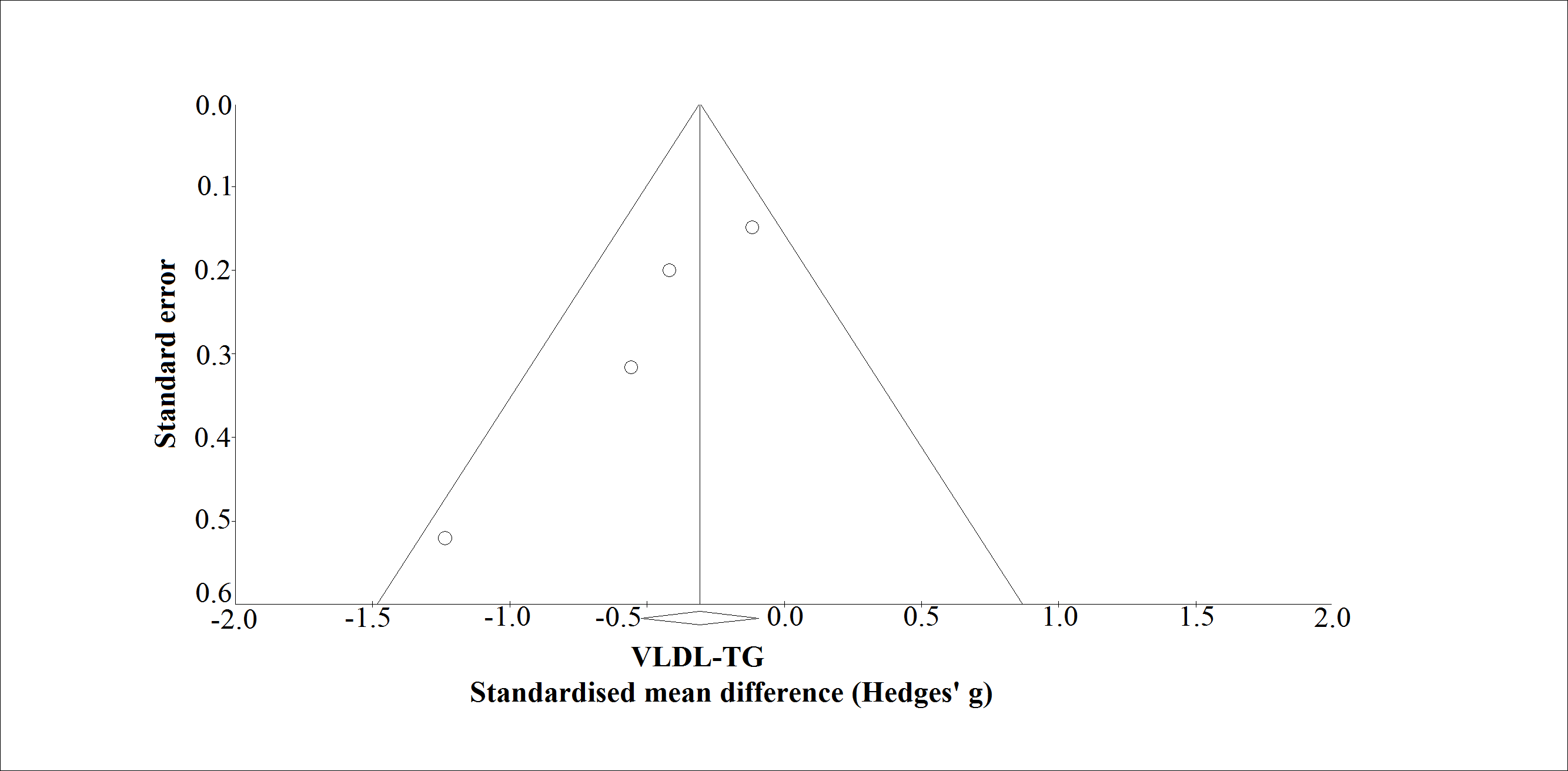

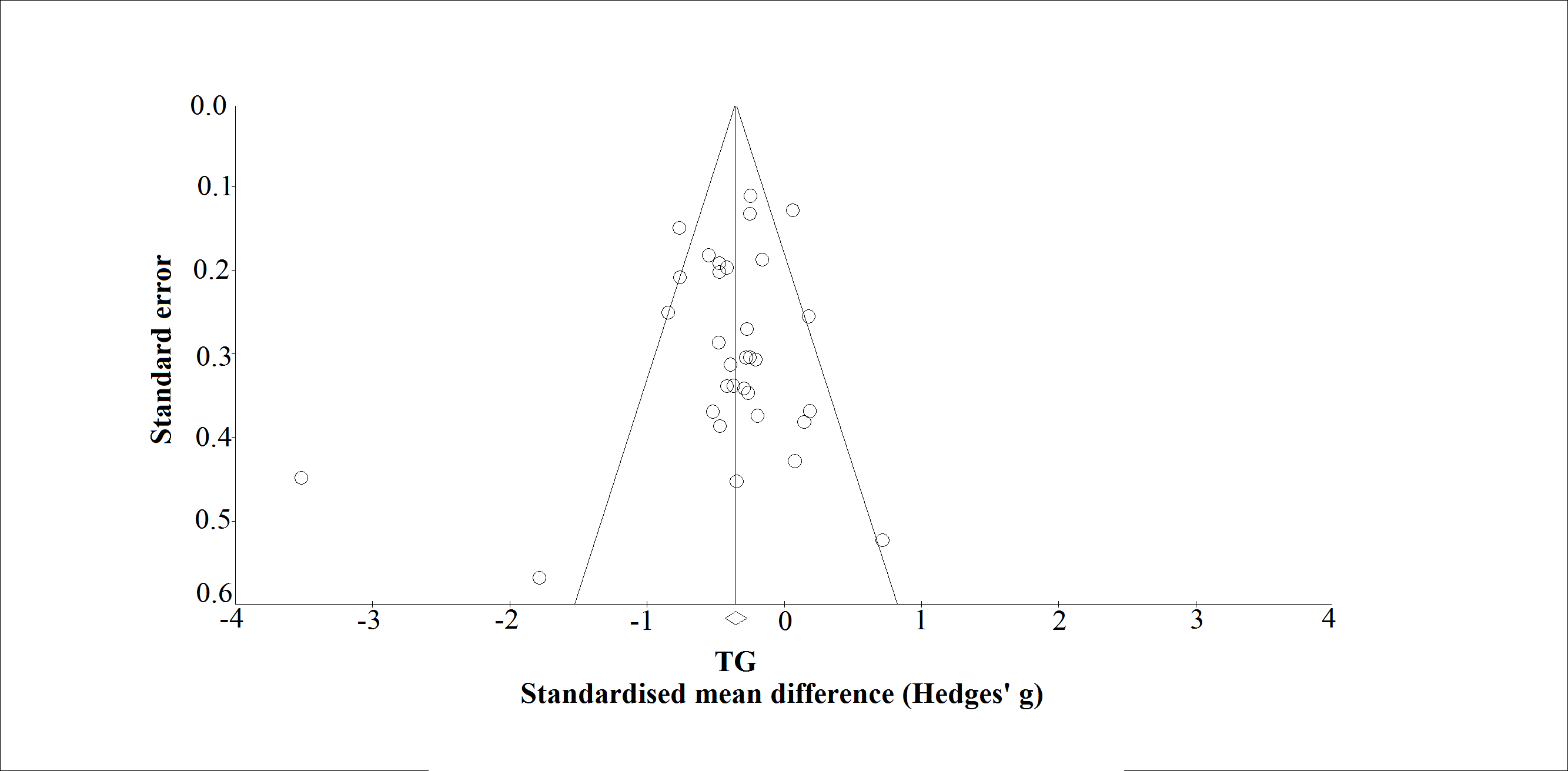

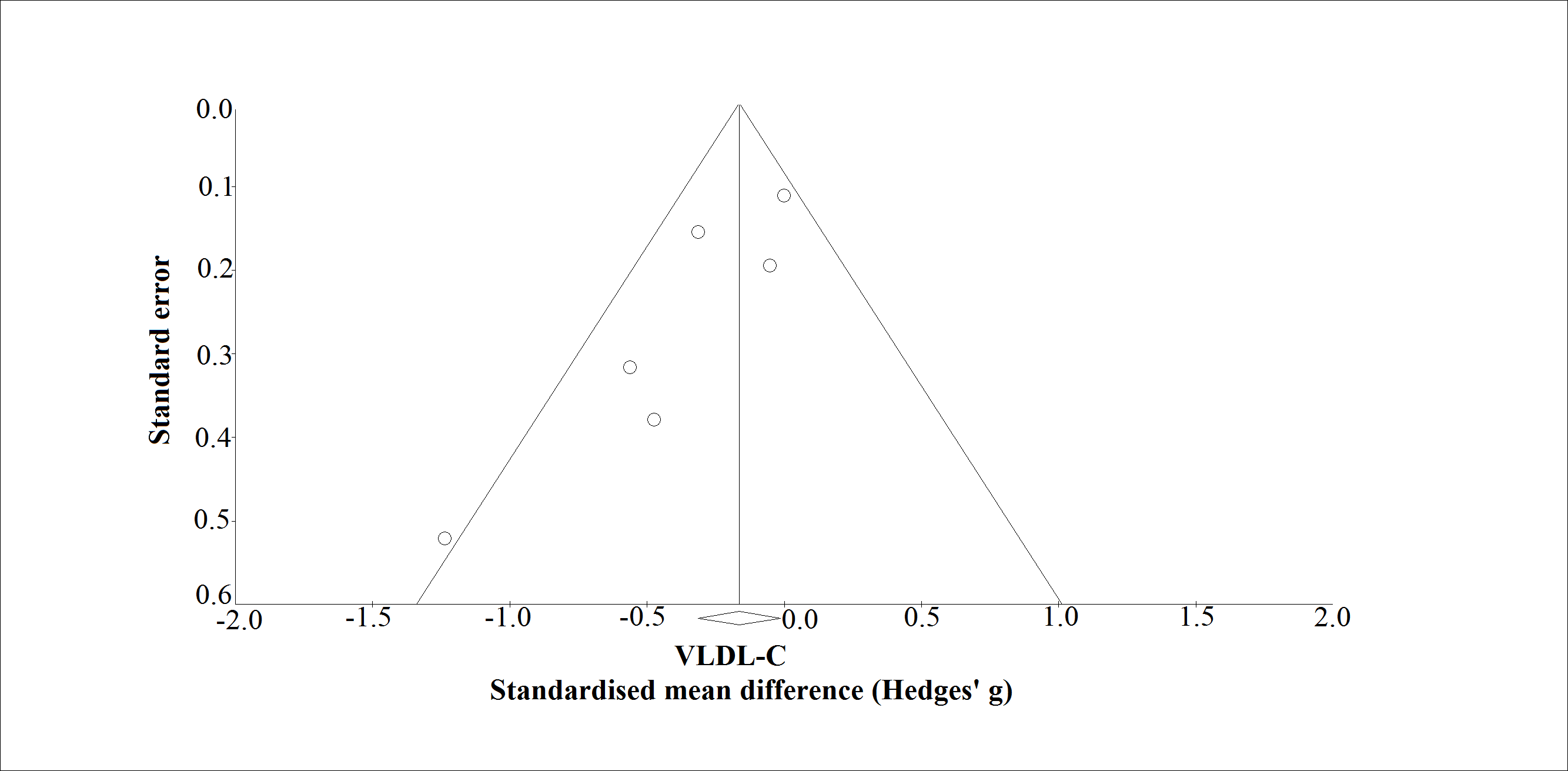

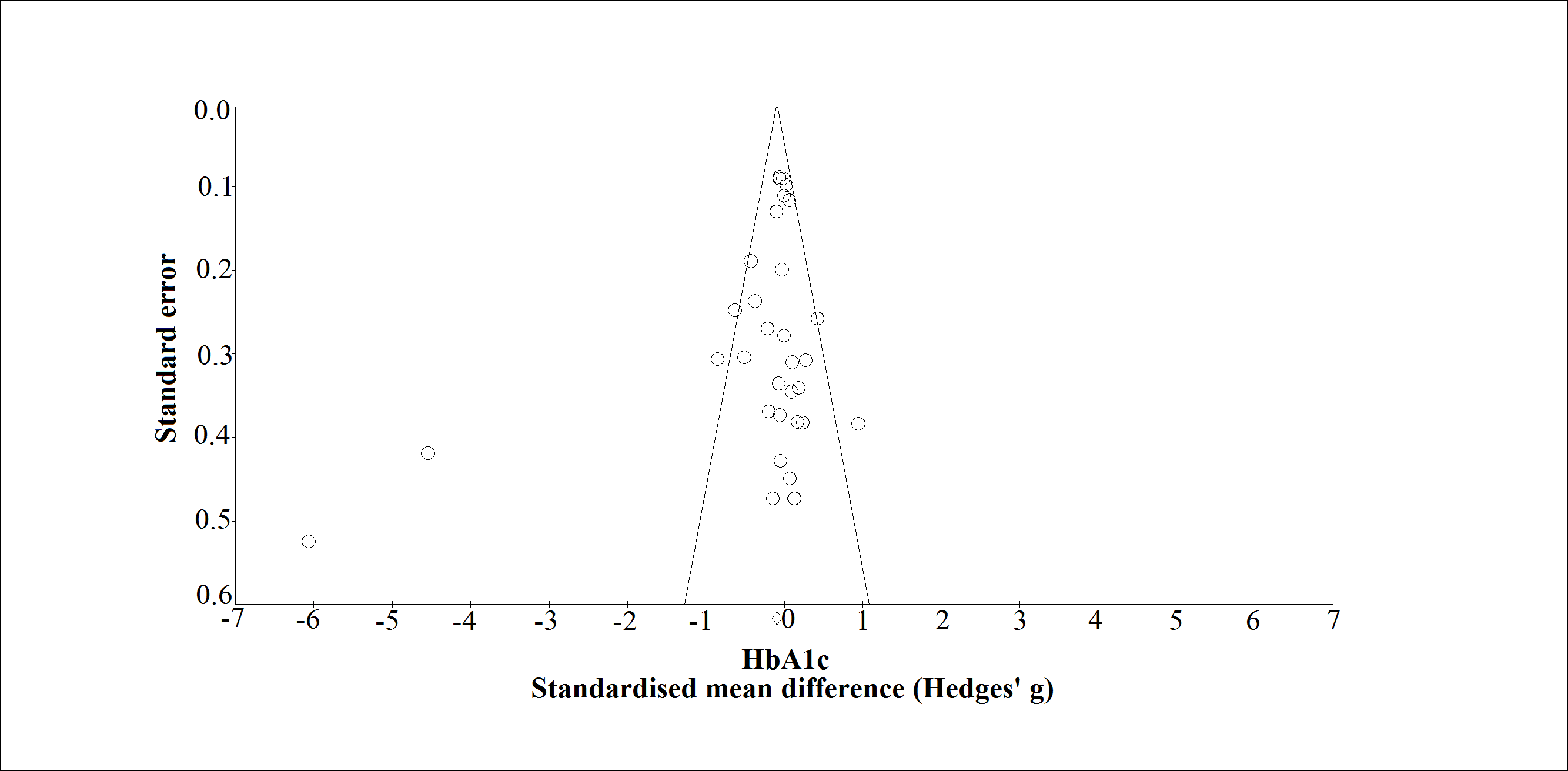

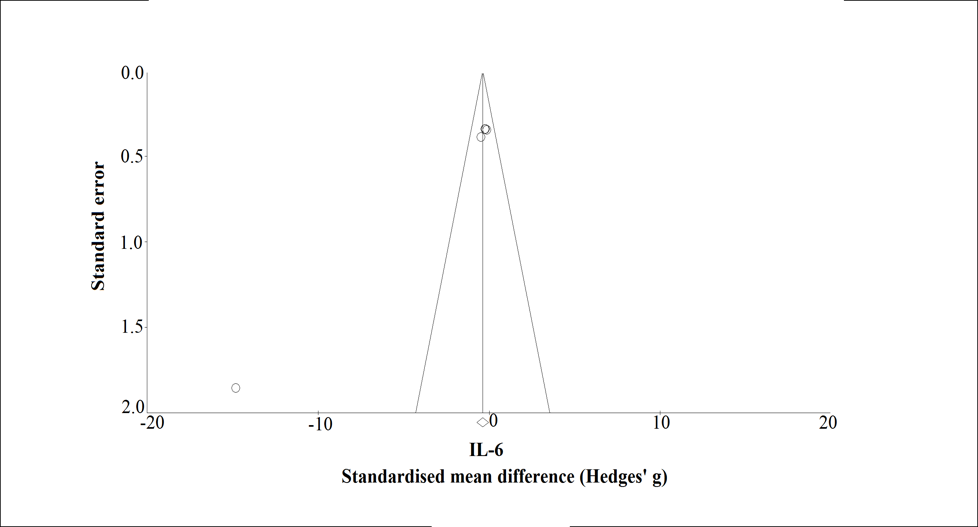

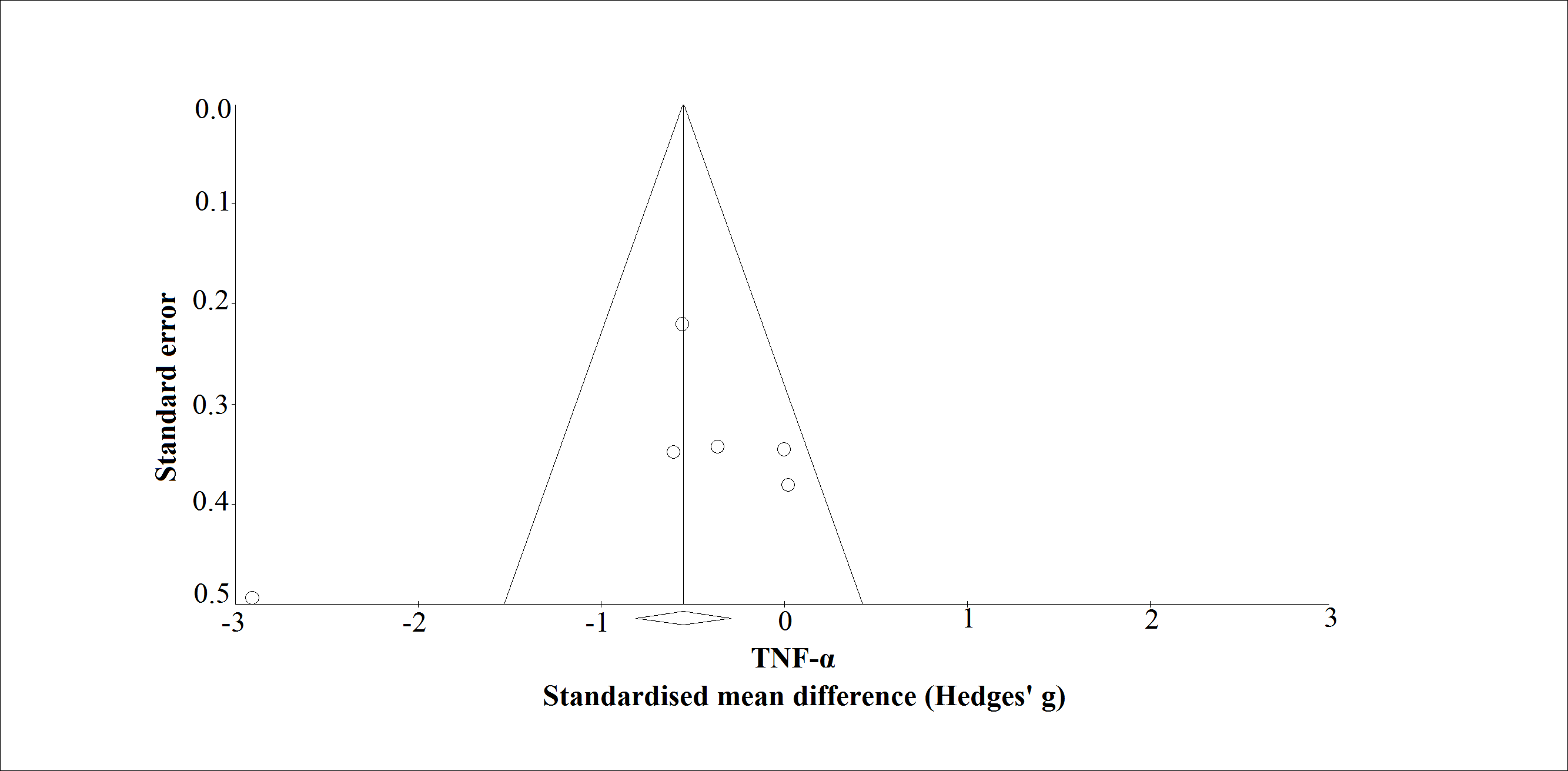

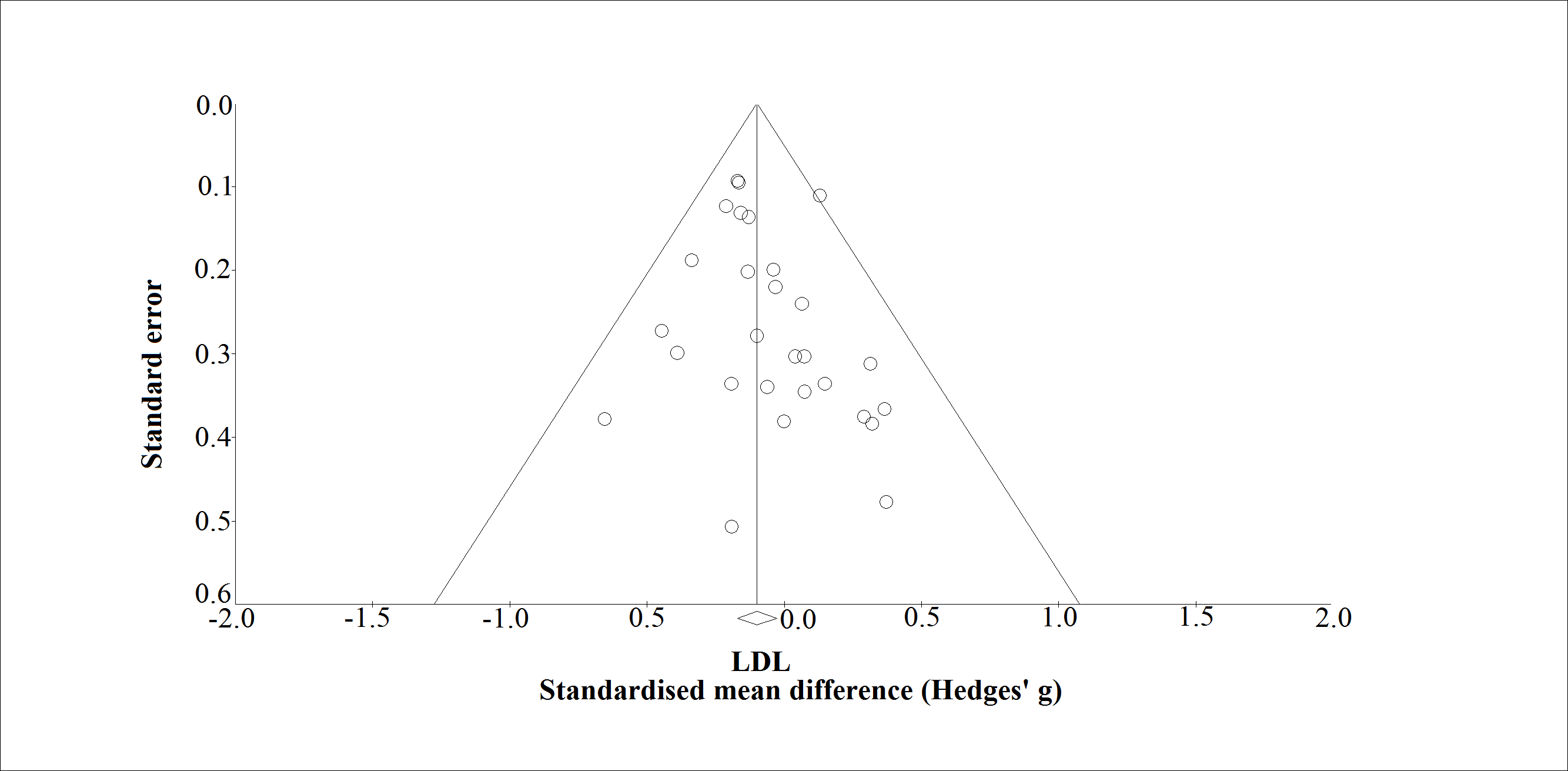


F

E

D

B

G

A

C

**Figure S3 A-G.** Funnel plot of standard error by standard difference in means for LDL (A), TG (B), VLDL-C (C), VLDL-TG (D), TNF-α (E), IL-6 (F), and HbA1c (G). LDL, low density lipoprotein cholesterol; TG, triglycerides; VLDL-C, very low density lipoprotein cholesterol; VLDL-TG, very low density lipoprotein triglycerides; TNF-α, tumour necrosis factor alpha; IL-6, Interleukin 6; HbA1c, glycated haemoglobin.
